# Amino-Functionalized DWCNTs Tailor Curing Kinetics and Multifunctional Performance of Epoxy Nanocomposites

**DOI:** 10.3390/polym18172087

**Published:** 2026-08-28

**Authors:** Raffaele Longo, Liberata Guadagno, Marialuigia Raimondo, Francesca Aliberti, Michelina Catauro, Luigi Vertuccio

**Affiliations:** 1 Department of Industrial Engineering, University of Salerno, Via Giovanni Paolo II, 132, 84084 Fisciano, SA, Italy; rlongo@unisa.it (R.L.); lguadagno@unisa.it (L.G.); mraimondo@unisa.it (M.R.); faliberti@unisa.it (F.A.); 2 Department of Engineering, University of Campania “Luigi Vanvitelli”, Via Roma 29, 81031 Aversa, CE, Italy; michelina.catauro@unicampania.it

**Keywords:** epoxy nanocomposites, functionalized carbon nanotubes, isoconversional DSC, electrical percolation, glass transition temperature

## Abstract

The influence of amino-functionalized double-walled carbon nanotubes (DWCNTNH_2_) on the curing behavior and multifunctional properties of an epoxy resin was systematically investigated. Isothermal FTIR analysis, interpreted using Kamal’s autocatalytic model and model-free isoconversional DSC analysis, showed that the presence of amino-functionalized nanotubes accelerates the initial epoxy curing reaction, increasing the primary reaction rate constant, reducing the activation energy, and confirming the catalytic role of the nanotube surface amino groups. Thermal–mechanical analysis indicated the formation of an interphase characterized by locally reduced crosslink density resulting from reactions between the functionalized nanotubes and the epoxy precursor. This interphase slightly lowers the glass transition temperature and the onset of thermal degradation without significantly affecting the overall thermomechanical performance. The incorporation of the filler also produces a remarkable increase in electrical conductivity, with an electrical percolation threshold between 0.1 and 0.3 wt%. Conversely, moisture diffusion and equilibrium water uptake remain essentially unchanged, demonstrating that the low nanotube content does not significantly alter the diffusion pathways or the polarity of the crosslinked network.

## 1. Introduction

The integration of nanostructured fillers into polymer matrices has emerged as a well-established route for engineering material properties according to the demands of targeted applications. In comparison with neat polymers, nanocomposite systems commonly display superior mechanical strength, electrical conductivity, thermal transport, and multifunctional characteristics [1,2,3,4]. These property enhancements can be achieved either by modifying the molecular structure of the polymer [5] or by introducing reinforcing nanofillers capable of imparting functionalities that are not present in the pristine matrix [6,7,8,9,10,11]. This strategy has been extensively explored for epoxy resins, owing to their widespread use as structural adhesives, protective coatings, encapsulation materials for electronic devices, and matrix materials in fiber-reinforced composites employed in aerospace, marine, and automotive applications [12,13,14,15]. Among the numerous application areas, electrically conductive epoxy nanocomposites have attracted increasing interest because of the growing demand for materials combining efficient electrical transport with effective thermal management in advanced electronic and semiconductor technologies [16,17,18,19]. Carbon-based nanomaterials, such as carbon nanotubes (CNTs), graphite, graphene, and their derivatives, are considered particularly attractive reinforcing agents due to their exceptional intrinsic electrical and thermal conductivities, which can be transferred to the polymer matrix [20,21,22,23]. Conventional epoxy resins typically possess an electrical conductivity on the order of 10^−9^ S/m [24] and a thermal conductivity between 0.17 and 0.21 W/mK [25]. In contrast, CNTs exhibit electrical conductivities of approximately 10^6^–10^7^ S/m [26] and 10^8^ S/m [26], respectively, while their thermal conductivities reach approximately 2800–6000 W/mK [27] and 1500–5000 W/mK [28]. Consequently, the incorporation of suitable amounts of these nanofillers offers an effective route to substantially improve the electrical and thermal transport capabilities of epoxy-based materials. Numerous investigations have confirmed the effectiveness of carbon nanofillers in enhancing epoxy performance. Fogel et al. [29], for instance, prepared epoxy/multiwalled carbon nanotube nanocomposites by means of three-roll milling and observed an increase in electrical conductivity spanning approximately eleven orders of magnitude. Their study identified a percolation threshold of 0.25 wt% CNT, while the highest conductivity achieved was 0.006 S/m at a nanotube content of 0.75 wt%. Using the same processing approach, Imran et al. [30] fabricated graphene-reinforced epoxy nanocomposites and reported that the addition of 2 wt% graphene increased the electrical conductivity to 3.1 × 10^−4^ S/m. They also found that the thermal conductivity rose from 0.14 W/mK for the unfilled epoxy to 0.27 W/mK when 1 wt% graphene was incorporated. Besides enhancing the functional characteristics of epoxy resins, nanofillers can significantly influence the curing process. The presence of nanoscale reinforcements modifies the processing behavior of the resin relative to the neat system, making a detailed understanding of the curing mechanism and reaction kinetics essential for process optimization and for maximizing the final properties of the composite. For this reason, the curing behavior of epoxy systems has been extensively investigated. Differential scanning calorimetry (DSC) has been the most widely adopted technique for kinetic analysis, with reported activation energy values ranging from 28 to 158 kJ/mol for unfilled epoxy formulations [31,32]. Although these values vary considerably depending on the formulation and analysis method, several studies have shown that the activation energy may remain nearly constant over a large portion of the curing process [33,34]. The effect of carbon nanofillers on epoxy cure kinetics has likewise received considerable attention [35,36,37,38,39]. Xie et al. [37,38,39] carried out a systematic investigation on epoxy nanocomposites reinforced with multiwalled carbon nanotubes and carbon nanofibers. Their results demonstrated that multiwalled carbon nanotubes promoted the initial stages of curing, leading to progressively shorter times to reach the maximum reaction rate as the nanotube concentration increased [39]. They also observed a reduction in the activation energy associated with the curing reaction as the nanotube loading increased. By comparison, carbon nanofibers produced only limited changes in both the initial curing rate and the time required to attain the peak reaction rate [37,38]. Abdalla et al. [40] further evaluated the influence of chemically modified multiwalled carbon nanotubes by comparing fluorinated and carboxyl-functionalized nanotubes. While fluorinated nanotubes produced negligible changes in activation energy and reaction rate constants, carboxyl-functionalized nanotubes increased the activation energy and reduced the cure rate constants, indicating a slower curing process. Although carbon nanofillers generally improve the functional and mechanical characteristics of epoxy matrices, their presence may also influence the durability of the resulting materials under environmental exposure. Liu et al. [41] showed that immersion in distilled water for 60 days caused the tensile strength of neat epoxy to decrease by 42%, whereas graphene oxide-reinforced epoxy exhibited a much smaller reduction of only 6%, demonstrating the beneficial effect of graphene oxide against moisture-induced degradation. Similarly, Glaskova-Kuzmian et al. [42] reported reductions in flexural modulus of 29%, 21%, 18%, and 6% for neat epoxy, carbon nanotube/epoxy, glass fiber-reinforced epoxy, and carbon nanotube/glass fiber-reinforced epoxy composites, respectively, after exposure to humid conditions. These observations suggest that the nature and concentration of the incorporated nanofiller strongly influence both moisture absorption and the consequent degradation of mechanical properties. The effect of nanofillers on moisture uptake, however, is not univocal. Zulfli et al. [43] found that the equilibrium moisture absorption of carbon nanotube/glass fiber-reinforced epoxy composites increased with nanotube content. Specifically, the incorporation of 0.5 wt%, 1 wt%, and 1.5 wt% CNTs resulted in increases of 12%, 22%, and 44%, respectively, in the equilibrium moisture content relative to the corresponding glass fiber-reinforced epoxy composite. In contrast, graphene oxide has been reported to reduce the equilibrium moisture uptake of epoxy systems [41]. Accordingly, depending on the chemistry, morphology, and concentration of the nanofiller, epoxy nanocomposites may exhibit either higher [43,44] or lower [41,42,45,46] moisture absorption than the unmodified resin. Since moisture ingress directly affects the long-term durability and service performance of structural composites, reliable prediction of moisture diffusion is essential for accurate lifetime assessment and engineering design.

Based on these considerations, the present work investigates the influence of amino-functionalized double-walled carbon nanotubes on both the curing behavior and the final performance of an epoxy matrix. Particular attention is devoted to evaluating the effects of these nanofillers on cure kinetics, as well as on the electrical, thermomechanical, and humidity-sensing properties of the resulting nanocomposites. Although non-functionalized carbon nanotubes provide the best electrical properties in the resulting nanocomposites [47,48], -NH_2_-functionalized double-walled carbon nanotubes (DWCNTs) were selected because they exhibited the best electrical performance among the functionalized nanotubes investigated in our previous studies using the same resin system, based on diglycidyl ether of bisphenol A (DGEBA) and 4,4′-diaminodiphenylsulfone (DDS) [47,48].

## 2. Materials and Methods

### 2.1. Material and Preparation Procedure

The components of the epoxy matrix are described in Table 1.

The epoxy formulations were prepared using diglycidyl ether of bisphenol A (DGEBA) as the epoxy precursor and 4,4′-diaminodiphenylsulfone (DDS) as the curing agent, with a precursor-to-hardener weight ratio of 10:2.85. Resin components were supplied by Sigma Aldrich Missouri, USA. Amino-functionalized double-walled carbon nanotubes (DWCNTNH_2_, 2152 Grade) were supplied by Nanocyl S.A (Sambreville, Belgium). The amount of grafted amino groups, determined by X-ray photoelectron spectroscopy (XPS), was less than 0.5 wt%. The morphological and dimensional characteristics of the DWCNTNH_2_ used in this study have been previously reported [49]. A more complete description is shown in Table S1, and the TEM image is reported in Figure S1.

**Figure 1:**
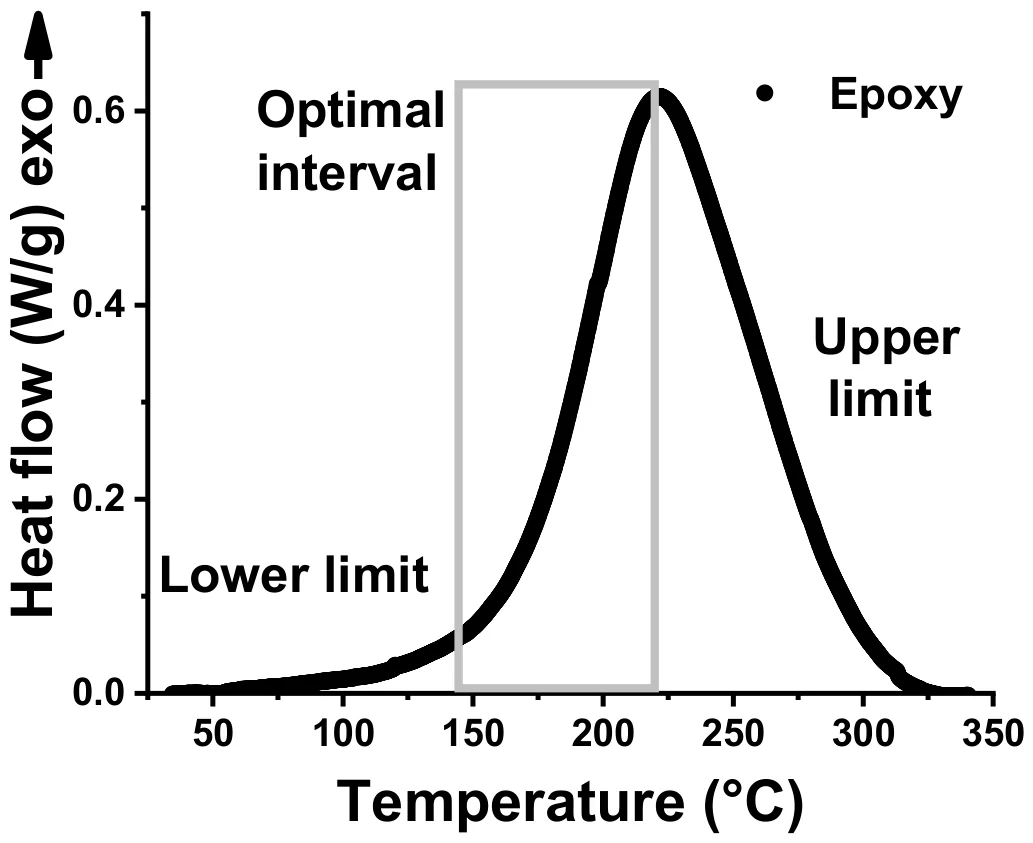
Selection of isothermal curing temperature.

The carbon nanotubes were first dispersed into the epoxy precursor by ultrasonic treatment using a Hielscher UP200S sonicator (Hielscher Ultrasonics, Teltow, Germany) operating at 24 kHz for 20 min. Subsequently, the nanotube/epoxy suspension was combined with the DDS hardener and homogenized by mechanical stirring at 120 °C for 1 h to ensure uniform mixing and adequate dispersion of the reinforcing phase. The curing procedure was defined through a two-step thermal cycle. Before selecting the isothermal curing temperatures, a dynamic DSC analysis was performed at a heating rate of 10 °C min^−1^ to identify the characteristic onset region of the curing reaction (Figure 1).

The curing temperatures were selected slightly above the onset temperature of the exothermic reaction to ensure effective crosslinking while avoiding excessively low temperatures, which could result in incomplete curing, or excessively high temperatures, which could promote undesired thermal effects. The selected temperature range (150–220 °C) falls within the optimal interval for the curing of the investigated epoxy system. Therefore, all samples were subjected to a first curing step at 150 °C for 1 h, followed by a post-curing treatment at 220 °C for 3 h. To evaluate the influence of nanotube content on the curing kinetics and final material properties, epoxy nanocomposites containing different DWCNTNH_2_ concentrations (0.1, 0.3, 0.5, and 1.0 wt%) were prepared and comparatively investigated in terms of kinetic behavior, electrical response, and mechanical performance.

### 2.2. Differential Scanning Calorimeter

Differential scanning calorimetry (DSC) measurements were performed using a Mettler DSC 822 instrument (Mettler-Toledo Columbus, OH, USA). Dynamic curing experiments and the subsequent kinetic analysis were carried out under a continuous nitrogen purge, maintained at a flow rate of 20 mL min^−1^, in order to provide an inert atmosphere and ensure reliable thermal measurements.

### 2.3. Electrical Measurements and Experimental Procedure

The electrical properties of the prepared nanocomposites were characterized by determining their direct-current (DC) volume conductivity using cylindrical specimens with a thickness of approximately 2 mm and a diameter of 50 mm. To ensure reproducible electrical contact and reduce the effects of surface roughness, both sides of each specimen were covered with a thin layer of silver conductive coating (≈50 μm), characterized by a surface resistivity of 0.001 Ω·cm. Electrical measurements were performed using a computer-controlled system based on the LAB-VIEW^®^ (2019) software platform. The experimental apparatus included a thermally regulated and electromagnetically shielded chamber, a Keithley 6517A electrometer (Keithley Instruments, Cleveland, OH, USA) used as both a high-voltage generator (up to ±1000 V) and a voltage measurement unit (up to ±200 V), and an HP34401A multimeter (Keysight Technologies, Santa Rosa, CA, USA) employed for current measurements with a minimum resolution of 0.1 μA in samples exhibiting electrical conductivity above the electrical percolation threshold (EPT). For nanocomposite specimens characterized by conductivity values below the EPT, the HP34401A instrument was replaced by the integrated picoammeter mode of the Keithley 6517A. This configuration enabled the detection of significantly lower currents, with a resolution down to 0.1 fA, while preserving the same voltage supply capability, thus allowing accurate electrical characterization across the entire conductivity range of the investigated materials.

### 2.4. Dynamic Mechanical Analysis

The dynamic mechanical behavior of the specimens was investigated using a dynamic mechanical thermal analyzer (DMA 2980, TA Instruments, New Castle, DE, USA). Rectangular specimens measuring approximately 4 × 10 × 35 mm^3^ were tested in the dual cantilever bending mode under oscillatory loading. A strain amplitude of 0.1% was applied throughout the experiments, while the testing frequency was maintained at 1 Hz. The measurements were carried out over a temperature interval ranging from 60 °C to 300 °C, using a constant heating rate of 3 °C min^−1^.

### 2.5. Thermogravimetric Analysis (TGA)

Thermogravimetric analysis (TGA) was performed using a Mettler TGA/SDTA 851 instrument (Mettler-Toledo Columbus, OH, USA). The measurements were conducted under an air atmosphere by heating the samples from 25 °C to 1000 °C at a constant heating rate of 10 °C min^−1^, to evaluate their thermal degradation behavior and stability.

### 2.6. FT/IR Analysis

Fourier transform infrared (FTIR) measurements were carried out using a BRUKER Vertex 70 spectrometer (Bruker Optics Inc., Billerica, MA, USA) equipped with a deuterated triglycine sulfate (DTGS) detector and a KBr beam splitter. The samples were analyzed using KBr pellets as the sampling medium. Spectra were collected with a resolution of 2.0 cm^−1^, and the wavenumber calibration was performed internally using a He–Ne laser, providing an accuracy of 0.01 cm^−1^. To enhance spectral quality and reduce random noise, each spectrum was generated by averaging 32 successive scans. The same FTIR system was also coupled with a temperature-controlled attenuated total reflectance (ATR) accessory (Golden Gate heated single-reflection diamond ATR, Specac-Teknokroma, Barcelona, Spain) to investigate the uncured epoxy formulations and to follow the evolution of the main reactive functional groups during the isothermal curing process at 180 °C. According to the selected experimental conditions, this temperature was adopted for monitoring the conversion of reactive species, with particular attention to the consumption of epoxy groups in both the neat epoxy formulation and the nanocomposite containing 0.5 wt% nanoparticles.

### 2.7. Water Sorption Analysis

Water sorption tests have been carried out according to procedures described in previous work [50,51].

### 2.8. Use of Generative Artificial Intelligence (GenAI)

Generative artificial intelligence (ChatGPT, GPT-5.6 Luna, OpenAI) was used solely to assist in the preparation of a schematic illustration included in Figure 9a, specifically the reaction scheme of the polyaddition process involving NH_2_-functionalized carbon nanotubes. The AI-generated content was reviewed, edited, and verified by the authors for scientific accuracy. No generative AI was used for data analysis, interpretation of results, or the preparation of the scientific conclusions.

## 3. Results and Discussion

### 3.1. Curing Behaviors (Isothermal FTIR Analysis)

**Figure 2:**
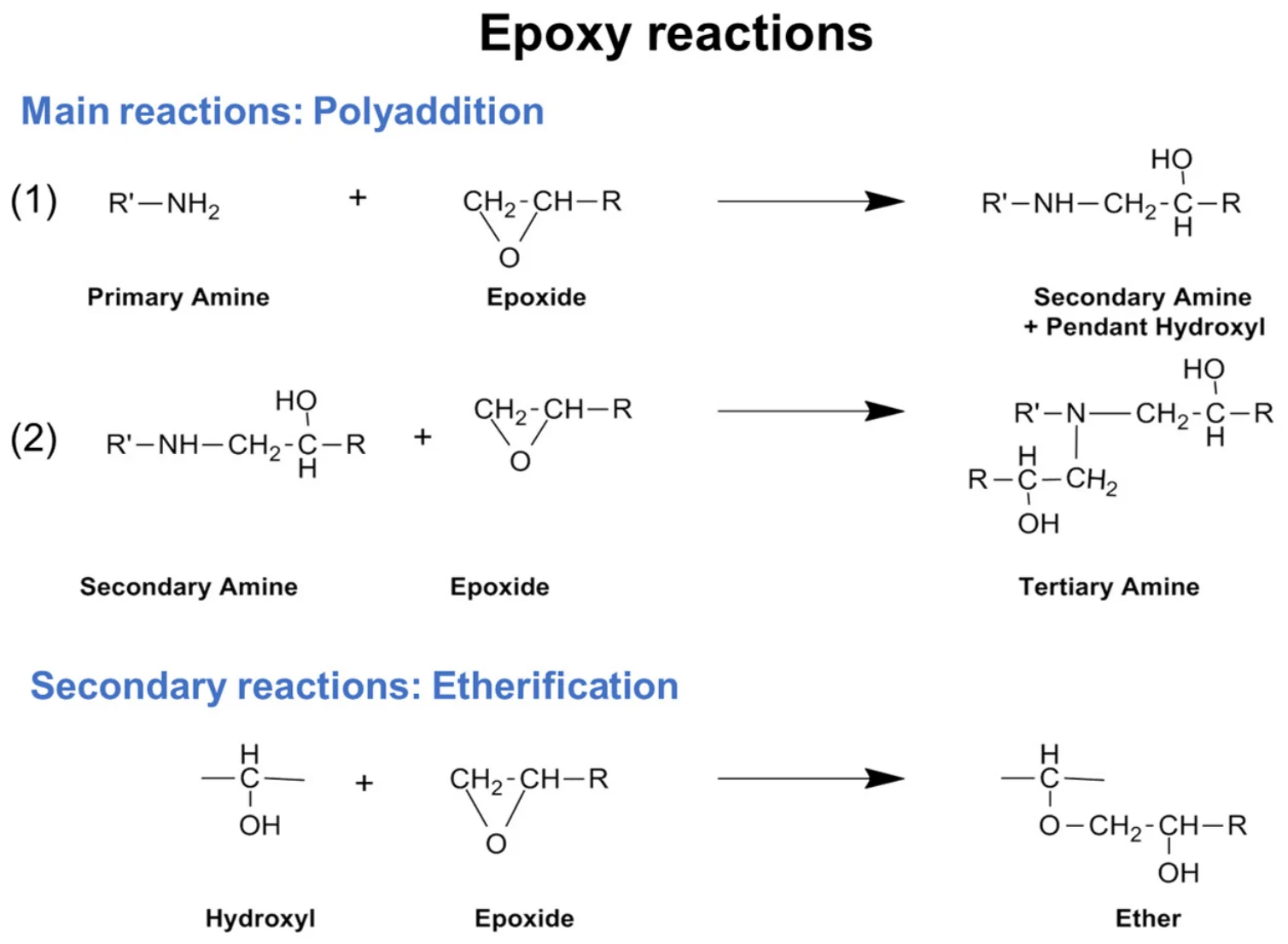
Schematic representation of the chemical pathways involved in the crosslinking process between epoxy precursor molecules and amine-based curing agents.

Fourier transform infrared (FTIR) spectroscopy was employed to investigate the evolution of the curing reactions during polymerization at 180 °C. The absorption band located at 913 cm^−1^, associated with the epoxy (oxirane) ring, was selected as the characteristic marker for monitoring the progress of the curing process. During epoxy–amine polymerization, crosslinking proceeds through the opening of the epoxy ring following its reaction with active hydrogen atoms of amine groups or with hydroxyl groups generated during curing through etherification, as schematically illustrated in Figure 2.

The curing mechanism involves three principal reaction pathways: (1) the addition of primary amines to epoxy groups, (2) the subsequent reaction of secondary amines with additional epoxy groups, and (3) etherification reactions. As the crosslinked network progressively develops, molecular mobility becomes increasingly restricted, leading to a transition from a kinetically controlled regime to one governed predominantly by diffusion. Etherification occurs through the reaction between hydroxyl groups formed during the initial curing stages and unreacted epoxy groups, resulting in the formation of ether linkages. In the presence of tertiary amines or other catalytic species, epoxy groups may also undergo homopolymerization. Under these conditions, the reaction preferentially yields linear polymer chains rather than contributing to the formation of the three-dimensional crosslinked network.

**Figure 3:**
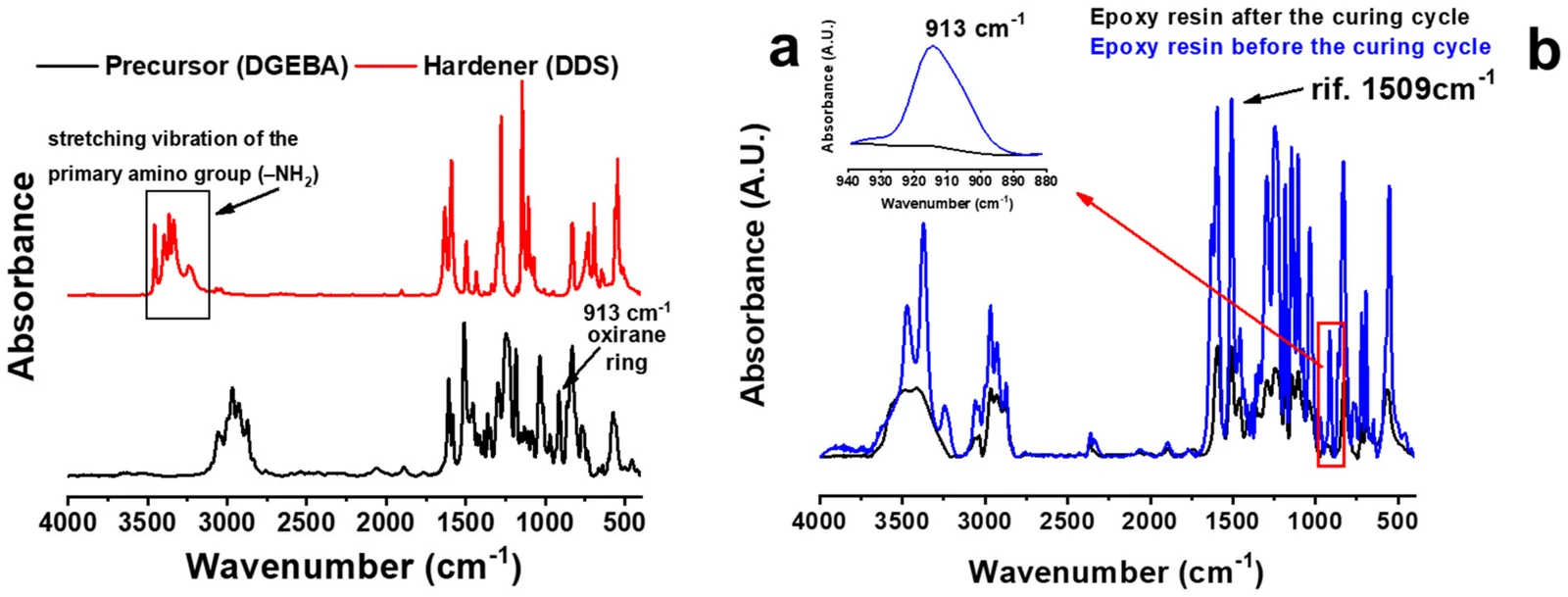
FTIR spectra of (**a**) the individual epoxy components (precursor and hardener) before curing; (**b**) epoxy resin formulation before and after the thermal curing cycle.

Figure 3a presents the FTIR spectra of the epoxy precursor and the amine curing agent before thermal curing, whereas Figure 3b reports the spectrum of the fully cured epoxy resin. The characteristic absorption bands of the precursor and the hardener are identified in Figure 3a. In particular, the epoxy precursor exhibits the characteristic oxirane-ring vibration at 913 cm^−1^, while the hardener displays three absorption bands at 3369, 3298, and 3174 cm^−1^, corresponding to the stretching vibrations of the primary amino (–NH_2_) groups. During curing, the reaction between epoxy groups and amine functionalities promotes the opening of the oxirane ring and the formation of hydroxyl groups, in agreement with the reaction scheme reported in Figure 2. The generation of hydroxyl species is evidenced in the FTIR spectrum of the cured resin (Figure 3b) by the broad absorption band extending from 3150 to 3670 cm^−1^. The advancement of the curing reaction was therefore evaluated by simultaneously monitoring the progressive decrease in the intensity of the epoxy-ring absorption at 913 cm^−1^ and the corresponding increase in the hydroxyl absorption band within the 3150–3670 cm^−1^ spectral region, as shown in Figure 3b.

**Figure 4:**
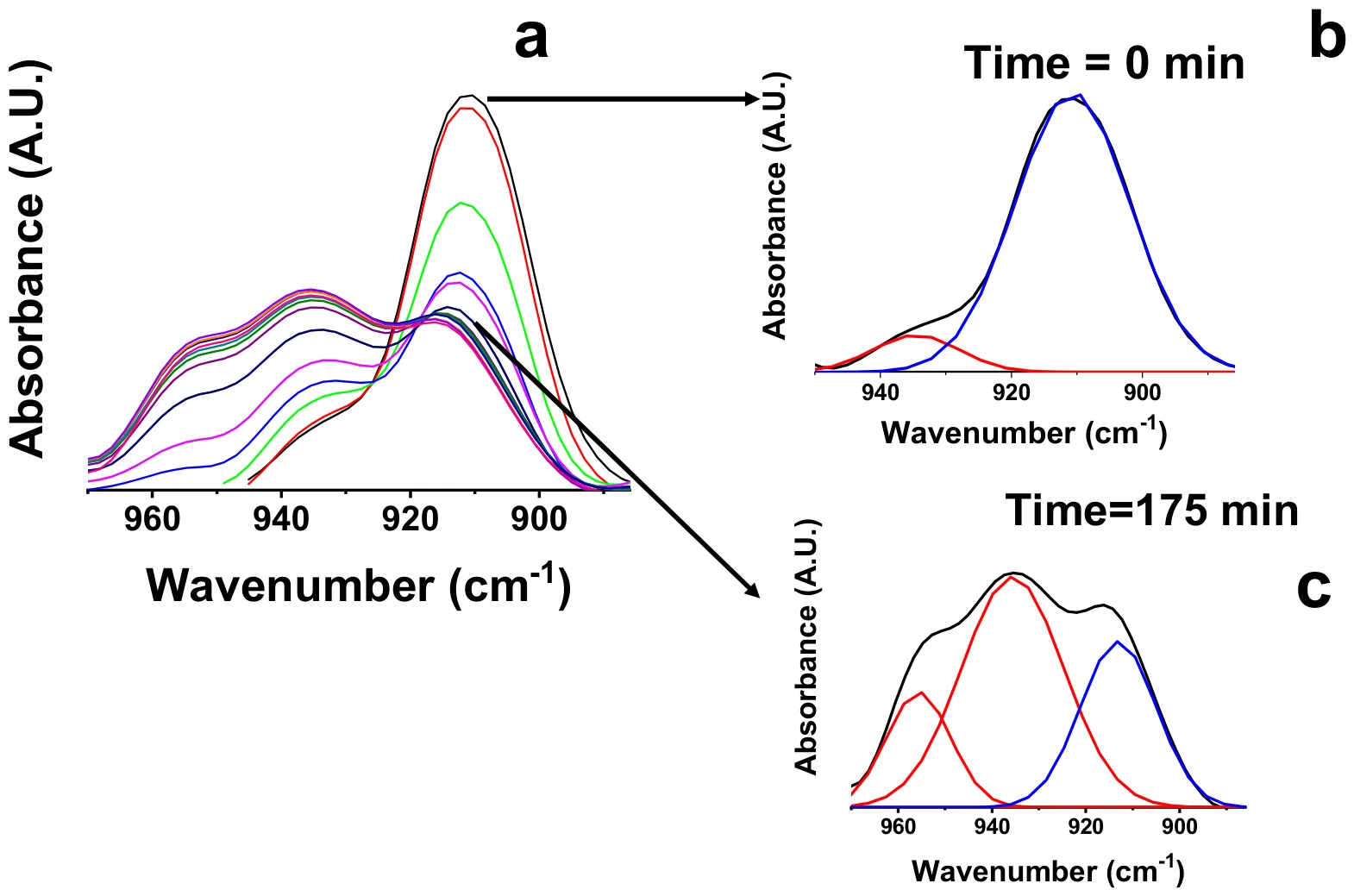
(**a**) Time evolution of the FTIR spectrum of the resin, in the range assigned to the epoxy ring at a temperature of 180 °C. Fitting of the peaks calculated by Equation 3 (**b**) at time = 0 min and (**c**) time = 175 min.

The extent of the curing reaction was quantified by determining the conversion of the reactive epoxy groups using the Lambert–Beer relationship:

(1) I=ε·c·l

where *I* represents the absorbance at the selected wavenumber, *ε* is the molar absorptivity, *c* is the concentration of the absorbing species, and *l* is the optical path length. The degree of cure was evaluated from the progressive consumption of the epoxy (oxirane) groups by monitoring the characteristic absorption band at 913 cm^−1^ according to:

(2) $$\alpha =\frac{I_{913;t=0}^{\prime }-I_{913;t}^{\prime }}{I_{913;t=0}^{\prime }-I_{913\;total\;curing}^{\prime }}$$

where *I′*_913_ = *I*_913_/*I*_1509_ denotes the normalized absorbance of the epoxy band. The absorption peak at 1509 cm^−1^, assigned to the aromatic phenyl ring of the epoxy precursor, was selected as an internal reference because it remains chemically unaffected throughout the curing reaction. Consequently, normalization with respect to this band compensates for possible variations in sample thickness and spectral acquisition conditions, providing a reliable measure of epoxy conversion. This normalization procedure has also been successfully adopted for other thermosetting systems [52,53]. The subscripts *t* = 0, *t*, and *total curing* correspond to the uncured resin, an intermediate curing time, and the fully cured material, respectively. The absorbance associated with complete conversion was determined from specimens subjected to the thermal curing cycle described in the Materials and Methods section, and the achievement of full cure was subsequently verified by differential scanning calorimetry (DSC). Because the absorption region associated with the epoxy ring (Figure 4a) consists of overlapping contributions, spectral deconvolution was carried out before quantitative analysis. The experimental spectra were fitted using a combination of Gaussian and Lorentzian functions. Resolution of the unresolved multicomponent bands was performed through a nonlinear optimization procedure based on the Levenberg–Marquardt algorithm [54]. To improve the robustness and uniqueness of the fitting procedure, the baseline, the line-shape function, and the number of spectral components were kept constant throughout the analysis. The minimum number of individual bands required for an accurate description of the experimental spectrum was initially estimated by inspecting changes in the slope of the measured profile. The fitting routine subsequently optimized the position, intensity, and full width at half maximum (FWHM) of each component through nonlinear least-squares minimization.

The individual absorption bands were described using a mixed Gaussian–Lorentzian function of the following form [55]:

(3) $$f(x)=\left(1-L\right)H\exp\left[-4\ln\left(2\right){\left(\frac{x-x_{0}}{w}\right)}^{2}\right]+LH{\left[4{\left(\frac{x-x_{0}}{w}\right)}^{2}+1\right]}^{-1}$$

where *x*_0_ = the peak position; *H* = peak height; *w* = FWHH; *L* = fraction of Lorentz character.

Figure 4 shows an example of the obtained fitting by applying Equation 3 for the pure resin system in the initial state (see Figure 4b) and after a cure cycle at 180 ° C for a time of 275 min (see Figure 4c).

**Figure 5:**
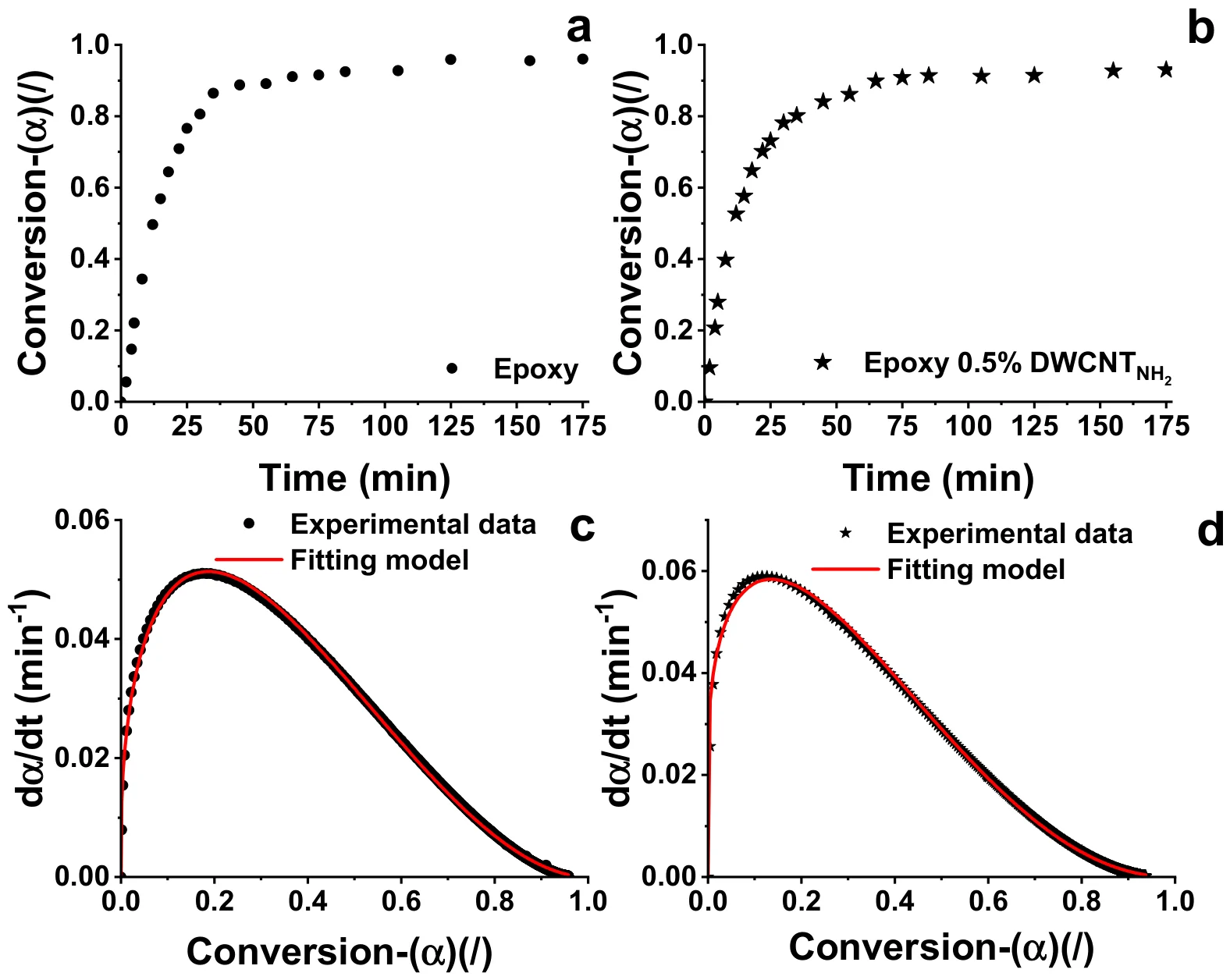
Isothermal conversion profiles as a function of curing time at 180 °C for (**a**) neat epoxy resin and (**b**) epoxy nanocomposite containing 0.5 wt% DWCNTNH_2_. Comparison between experimental conversion data (symbols) and values predicted by the kinetic model (solid red lines) for (**c**) neat epoxy resin and (**d**) epoxy resin filled with 0.5 wt% DWCNTNH_2_.

Figure 5a,b illustrate the evolution of the epoxy-ring conversion, determined from the decrease in the absorption band at 913 cm^−1^ according to Equation (2), for the neat epoxy resin and for the epoxy nanocomposite containing 0.5 wt% carbon nanotubes. Several kinetic models have been developed to describe the curing behavior of thermosetting polymers. In epoxy systems cured with amine hardeners, the reaction mechanism is generally considered to be governed by two successive epoxy ring-opening reactions involving primary and secondary amine groups [5,56]. In the present study, the isothermal curing behavior was analyzed using the autocatalytic model proposed by Kamal, which is commonly employed to describe chemically controlled curing processes [57]:

(4) $$\frac{d\alpha }{dt}=\left(k_{1}+k_{2}\alpha^{m}\right){\left(1-\alpha \right)}^{n}$$

where *m* and *n* are the reaction orders, and *k*_1_ and *k*_2_ are the rate parameters, which are functions of temperature. The kinetic parameters (*m*, *n*, and *k*_2_) were determined by nonlinear regression of the experimental conversion data. The value of *k*_1_ was obtained independently from the initial reaction rate, corresponding to the intercept at *t* = 0 of the reaction-rate-versus-time curves. As demonstrated in Figure 5c,d, the predictions provided by Equation (4) closely reproduce the experimental conversion-rate profiles over the entire range of cure, indicating that Kamal’s autocatalytic model accurately captures the curing behavior of the investigated systems. The kinetic parameters extracted from the fitting procedure for the amine-cured epoxy formulations are summarized in Table 2 together with the corresponding coefficients of determination, confirming the excellent agreement between the experimental measurements and the model predictions.

As reported in Table 2, both formulations exhibit an overall reaction order close to 2, while the kinetic parameter *k*_2_ remains essentially unchanged, with values of approximately 0.15 min^−1^ for both systems. A markedly different behavior is observed for *k*_1_, whose value in the carbon nanotube-reinforced epoxy is nearly one order of magnitude greater than that measured for the neat resin. According to Kamal’s kinetic model, *k*_1_ is associated with the reaction between primary amine groups and epoxy rings, as illustrated in Figure 2. Since Kamal’s model assumes that the activation energy does not vary with the degree of conversion, the higher *k*_1_ value suggests that the presence of carbon nanotubes accelerates the chemically controlled stage of the curing process. This enhancement is particularly evident during the early stages of crosslinking, indicating that the nanofiller mainly promotes the initial epoxy–amine reaction. Such behavior can be attributed to the amino functionalities located on the surface of the carbon nanotubes, which provide additional reactive sites capable of facilitating the opening of the epoxy rings and, consequently, increasing the initial reaction rate. Comparable effects have also been reported from both isothermal and dynamic calorimetric investigations of similar epoxy/carbon nanotube systems [58]. To further examine this catalytic effect, the curing behavior is subsequently analyzed under non-isothermal conditions by differential scanning calorimetry (DSC). Unlike the isothermal approach based on Kamal’s model, the dynamic analysis considers the activation energy as a conversion-dependent parameter, allowing the evolution of the crosslinking mechanism throughout the curing process to be evaluated in greater detail. The differences between isothermal and dynamic polymerization kinetics have been extensively discussed in the literature since the early development of kinetic modeling, and remain the subject of recent reviews. In general, these approaches should not be regarded as competing alternatives but rather as complementary methods. This complementary approach is particularly useful for complex epoxy systems, where chemical reaction kinetics, autocatalytic effects, molecular mobility, network formation, and vitrification can simultaneously contribute to the overall curing behavior.

### 3.2. Dynamic DSC Analysis (Isoconversional Methods and Model-Free Kinetics)

Changes in the activation energy throughout the curing process can provide important insights into the evolution of the underlying reaction mechanism. These variations are commonly evaluated using model-free isoconversional methods [59,60,61,62,63,64], in which the reaction rate at a given degree of conversion is related to the corresponding apparent activation energy according to:

(5) $${\left[\frac{d\;ln\left(d\alpha /dt\right)}{dt}\right]}_{\alpha }=\;-\frac{E_{\alpha }}{R}$$

Model-free isoconversional analyses are based on the principle that, for a fixed conversion level, the reaction rate is governed solely by the reaction temperature. Consequently, all quantities identified by the subscript (α) refer to values determined at the same extent of conversion, irrespective of the thermal history followed to reach that state. By applying this approach over the complete conversion range (0–1), it is possible to determine the apparent activation energy (*E_α_*) as a function of conversion, thereby providing a detailed description of the kinetic evolution of the curing process. For single-step processes, *E_α_* remains essentially constant throughout the reaction, whereas multistep mechanisms are characterized by a conversion-dependent activation energy, reflecting the changing contribution of the individual elementary reactions to the overall curing process. Among the available isoconversional techniques, the integral methods proposed by Sbirrazzuoli et al. [59] and further developed by Vyazovkin [65,66] are widely recognized because they employ numerical integration, thereby overcoming the limitations associated with conventional integral methods while accounting for possible variations in activation energy during the reaction.

In this study, the evolution of the apparent activation energy during epoxy curing was determined using the advanced isoconversional method proposed by Vyazovkin [65,66]. For a series of *n* experiments performed under different temperature programs (*Ti(t)*), the activation energy corresponding to a given degree of conversion is obtained by minimizing the following objective function:

(6) $$\sum_{i=1}^{n}\sum_{j\neq i}^{n}\frac{J\left[E_{\alpha },T_{i}\left(t_{\alpha }\right)\right]}{J\left[E_{\alpha },T_{j}\left(t_{\alpha }\right)\right]}$$

where the integral term is defined as

(7) $$J\left[E_{\alpha },T_{i}\left(t_{\alpha }\right)\right]\equiv \int_{t_{\alpha -\Delta \alpha }}^{t_{\varepsilon }}\exp\left[\frac{-E_{\beta }}{RT_{i}\left(t\right)}\right]dt$$

and is evaluated numerically for each experimental heating program. The integration is performed over small conversion intervals, as expressed by Equation (7), thereby minimizing the systematic errors typically associated with conventional integral approaches when the activation energy changes significantly with the extent of conversion. The conversion range is discretized from Δα to 1 − Δα using increments of Δα = m − 1, where *m* denotes the total number of conversion intervals adopted in the analysis. For experiments conducted under linear heating conditions, Equation (6) can be reformulated as:

(8) $$\sum_{i=1}^{n}\sum_{j\neq i}^{n}\frac{I\left[E_{\alpha },T_{\alpha ,i}\right]\beta_{j}}{I\left[E_{\alpha },T_{\alpha ,j}\right]\beta_{i}}$$

where b is the constant heating rate, and the integral function *I[E_α_, T_α,i_]* is evaluated using Doyle’s approximation [67]. The minimization procedure is carried out independently at each selected conversion level, allowing the apparent activation energy (*E_α_*) to be determined as a continuous function of the curing conversion. A major advantage of isoconversional techniques is that the activation energy can be evaluated without requiring prior knowledge of the pre-exponential factor. Since this kinetic parameter is not explicitly included in the calculation, the strong statistical dependence that frequently arises between the activation energy and the pre-exponential factor during conventional kinetic fitting procedures is avoided. As a result, the values of E_α_ obtained by the isoconversional approach are generally considered more robust and reliable [68].

**Figure 6:**
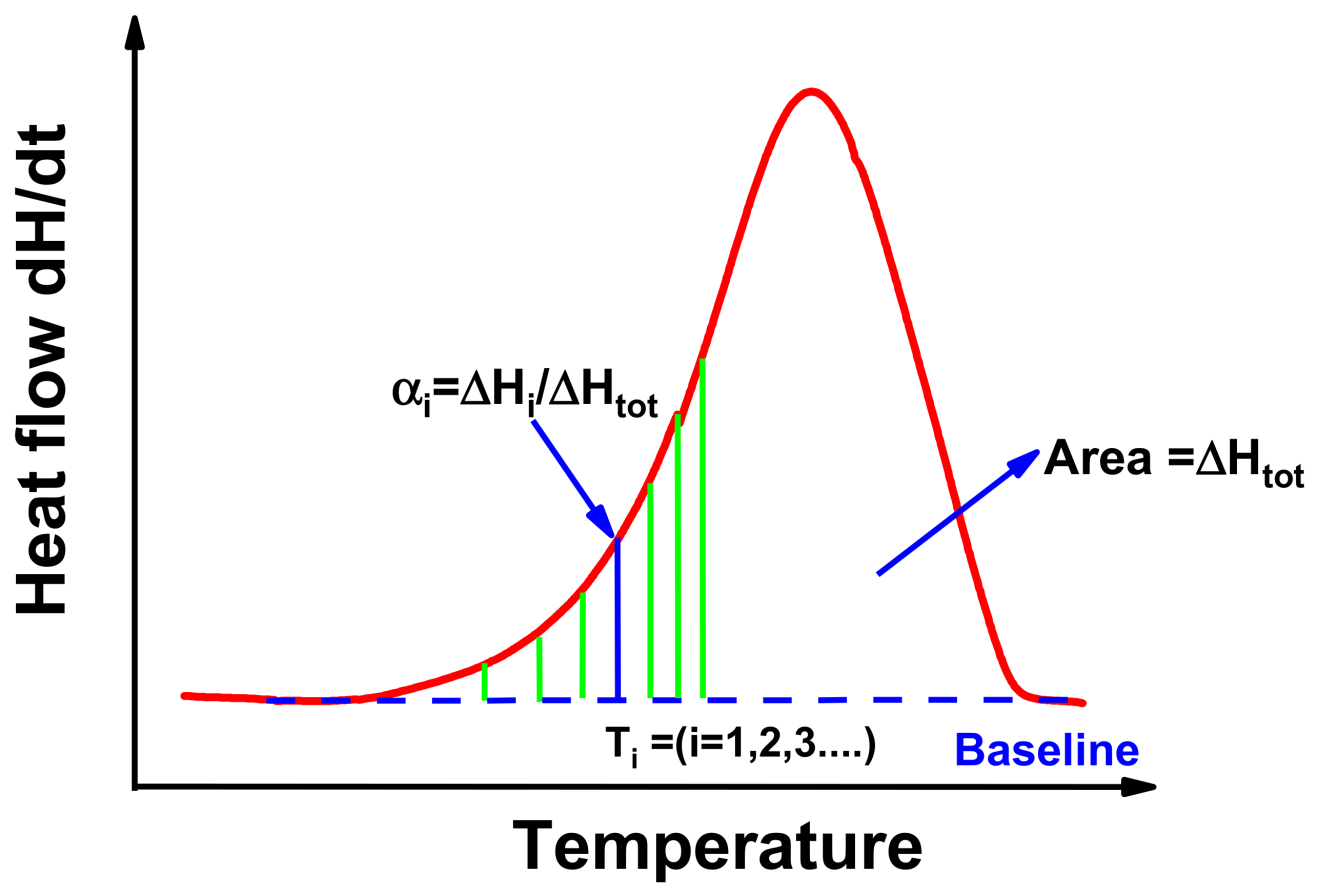
Representative DSC thermogram showing the characteristic exothermic peak associated with the curing reaction of an epoxy system.

**Figure 7:**
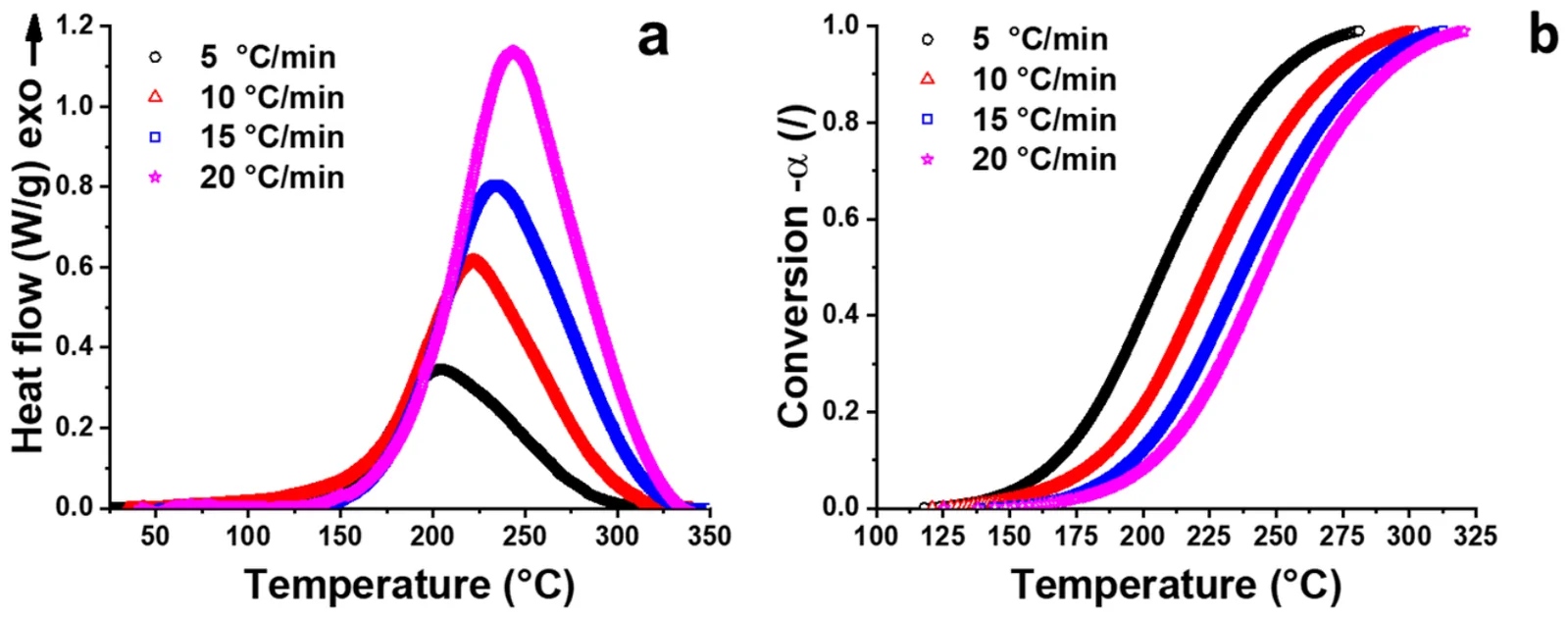
(**a**) Dynamic DSC thermograms of the neat epoxy resin recorded at heating rates of 5, 10, 15, and 20 °C min^−1^; (**b**) evolution of the degree of conversion (α) as a function of temperature for the epoxy resin measured under the same heating conditions.

In differential scanning calorimetry (DSC), the analysis of curing kinetics is based on the assumption that the heat generated by the crosslinking reaction is directly proportional to the reaction rate. Therefore, after correction for the instrumental baseline, the measured heat flow can be directly related to the progress of the curing process. Under dynamic heating conditions, however, the recorded signal contains not only the heat released by the chemical reaction but also the contribution associated with the heat capacity of the sample. To isolate the reaction heat, a suitable baseline is constructed beneath the exothermic peak corresponding to the curing event. A schematic representation of a typical DSC thermogram for an exothermic curing reaction is shown in Figure 6. For kinetic analysis, the degree of conversion at a given temperature is determined from the fraction of the total reaction enthalpy released during the curing process according to:

(9) $$\alpha \left(T,\beta \right)=\frac{\Delta H_{\beta }\left(T\right)}{\Delta H_{tot}}$$

where *ΔH_β_(T)* is the cumulative reaction enthalpy evolved up to temperature (T) at the heating rate (*β*), while *ΔH_tot_* represents the overall heat of reaction associated with complete curing. Dynamic DSC experiments were carried out at constant heating rates of 5, 10, 15, and 20 °C min^−1^ over the temperature interval from 30 to 300 °C. Representative DSC curves obtained for the neat epoxy formulation are presented in Figure 7a. The corresponding evolution of the degree of cure as a function of temperature, calculated using Equation (9), is reported in Figure 7b.

**Figure 8:**
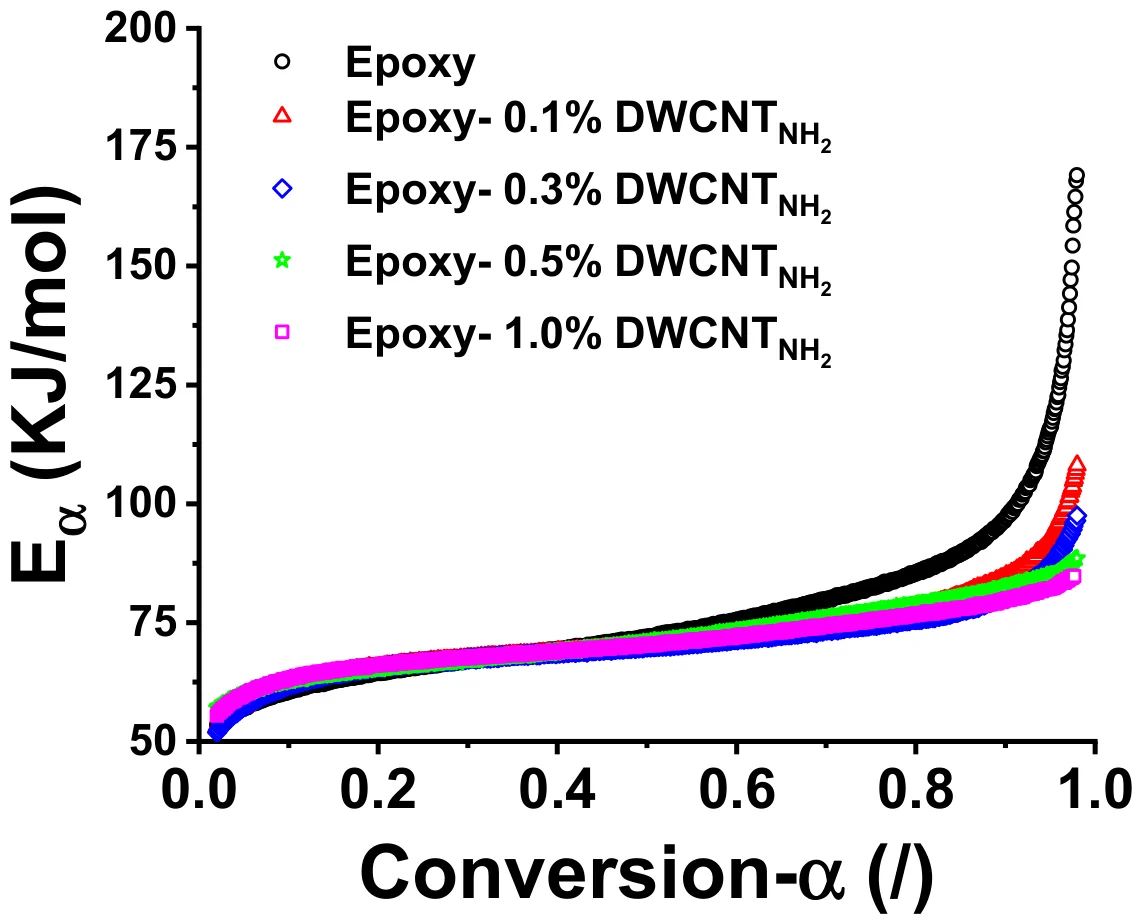
Dependence of the apparent activation energy on the degree of conversion for the neat epoxy resin and the carbon nanotube-reinforced composite systems.

Application of Equation (8) yielded the variation in *E_α_* with conversion, as illustrated in Figure 8. The *E_α_* increased progressively with the degree of cure, ranging from approximately 55 to 170 kJ/mol. This behavior is consistent with the progressive increase in viscosity during network formation, which restricts molecular mobility and requires progressively higher energy for chain segment rearrangement. As curing proceeds, the available free volume decreases, limiting the motion of the polymer chains to localized segmental movements. As curing progresses, the mobility of the reactive species gradually decreases because of the continuous formation of the crosslinked network. Consequently, the reaction becomes increasingly limited by diffusion, resulting in a progressive increase in the apparent activation energy during the final stages of the curing process.

**Figure 9:**
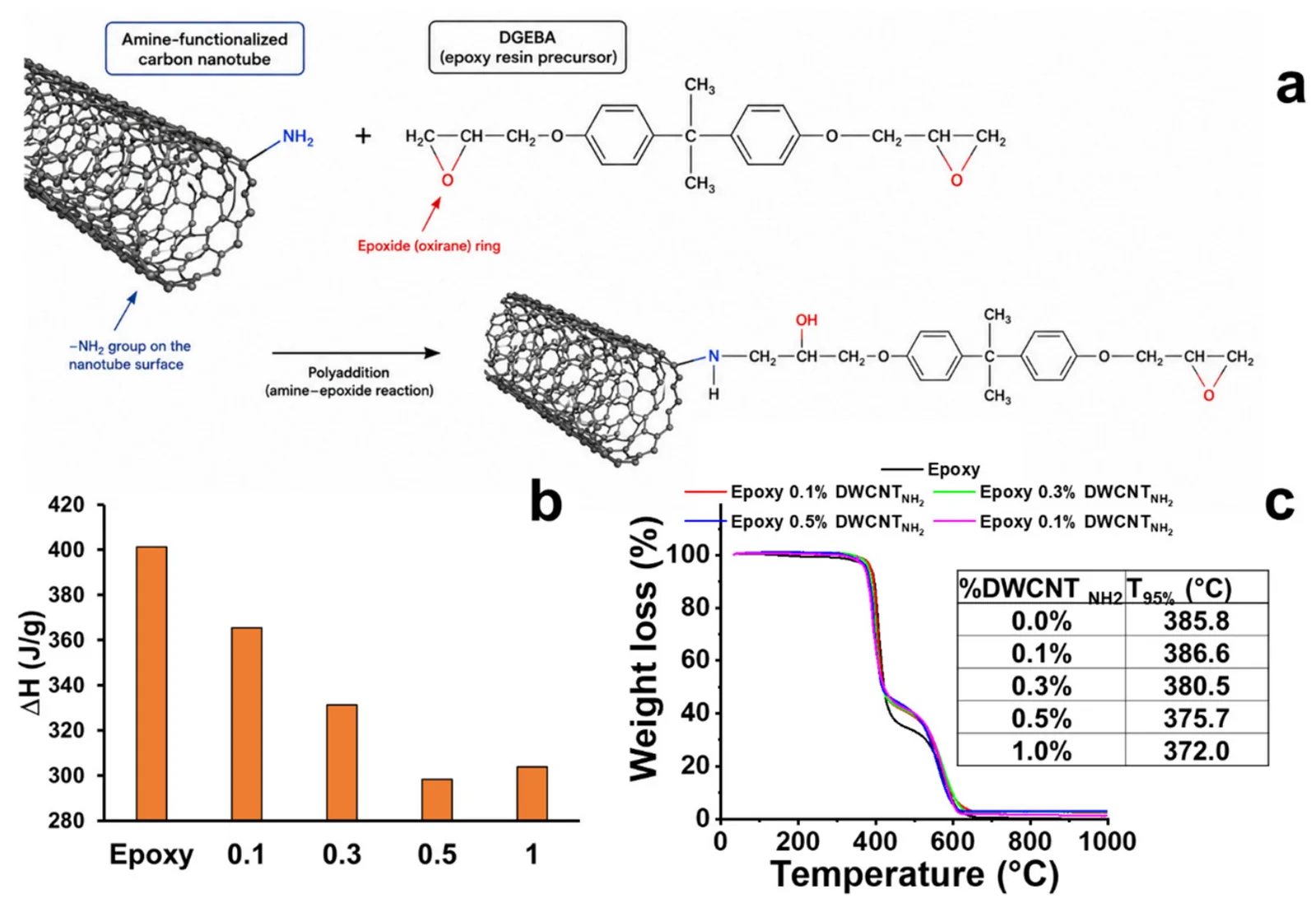
(**a**) Schematic representation of the reaction between functionalized DWCNTs and precursor resin; (**b**) reaction enthalpy (ΔH) of epoxy resin and composites; (**c**) thermogravimetric curve of epoxy resin and composites.

For all the investigated systems, the activation energy remains nearly constant up to a conversion of approximately 0.4. Beyond this conversion level, however, the nanocomposites exhibit lower activation energy values than the neat epoxy resin. Moreover, increasing the concentration of carbon nanotubes further reduces the apparent activation energy, particularly within the conversion interval where the reaction between epoxy groups and secondary amines predominates [56]. This behavior indicates that the presence of the nanofiller facilitates the progression of the curing reaction during its intermediate stages. In general, surface-functionalized carbon nanotubes containing polar functional groups, such as –COOH or –NH_2_, provide chemically active sites that promote the epoxy curing reaction, effectively accelerating the crosslinking process while producing only minor changes in the overall curing mechanism [58]. In our case, the catalytic effect is most likely associated with the functional groups present on the surface of the carbon nanotubes. Although the amino group content of the nanotubes is relatively low on a weight basis, these groups react with the epoxy precursor, as illustrated in Figure 9a, forming, most likely, an interphase with reduced molecular mobility around the nanotubes.

The formation of an interphase surrounding the functionalized carbon nanotubes introduces localized modifications to the curing process, partially altering the crosslinking reactions occurring in the surrounding epoxy matrix [5]. As a result, the overall heat released during polymerization decreases, as shown in Figure 9b. In particular, the total reaction enthalpy (ΔH) progressively declines as the concentration of amino-functionalized carbon nanotubes increases. This decrease can be attributed to a lower degree of polymerization, which is reflected in a reduction in the T_g_ value, as discussed in the following section. Additional evidence of the interaction between the amino functionalities grafted onto the nanotube surface and the epoxy precursor is provided by thermogravimetric analysis (TGA). The thermograms reported in Figure 9c compare the thermal degradation behavior of the neat epoxy resin with that of the nanocomposites containing different loadings of functionalized carbon nanotubes. Although all materials exhibit the characteristic two-step degradation pattern typical of epoxy systems, increasing the nanotube content causes the onset of degradation to shift toward lower temperatures. This trend becomes evident by considering the degradation onset temperature (T_95%_), defined as the temperature corresponding to a 5% mass loss. The value of (T_95%_) gradually decreases from approximately 386 °C for the unfilled epoxy to about 372 °C for the nanocomposite containing 1 wt% carbon nanotubes. Several factors may contribute to this behavior. The chemical functionalization required to graft amino groups onto the nanotube surface inevitably introduces structural defects within the graphitic framework. These defects enhance the chemical reactivity of the nanotubes [69] and may facilitate localized degradation phenomena or radical-induced reactions within the surrounding polymer network. Considering that the T_95%_ of the functionalized nanotubes alone is 490 °C (see Figure S2 of the Supplementary Materials), the observed decrease is not attributable to structural defects within the graphitic framework. Probably, the reaction between the surface –NH_2_ groups and the epoxy rings during curing promotes the formation of a strong polymer–nanotube interface. At the same time, this interfacial region is characterized by a lower local crosslink density than the bulk matrix. Consequently, degradation is initiated at lower temperatures, leading to a reduction in the initial thermal stability of the nanocomposite.

### 3.3. Mechanical Properties

Dynamic mechanical analysis (DMA) experiments in dual cantilever mode were performed to assess the mechanical properties of the epoxy resin and the composites at different temperatures.

**Figure 10:**
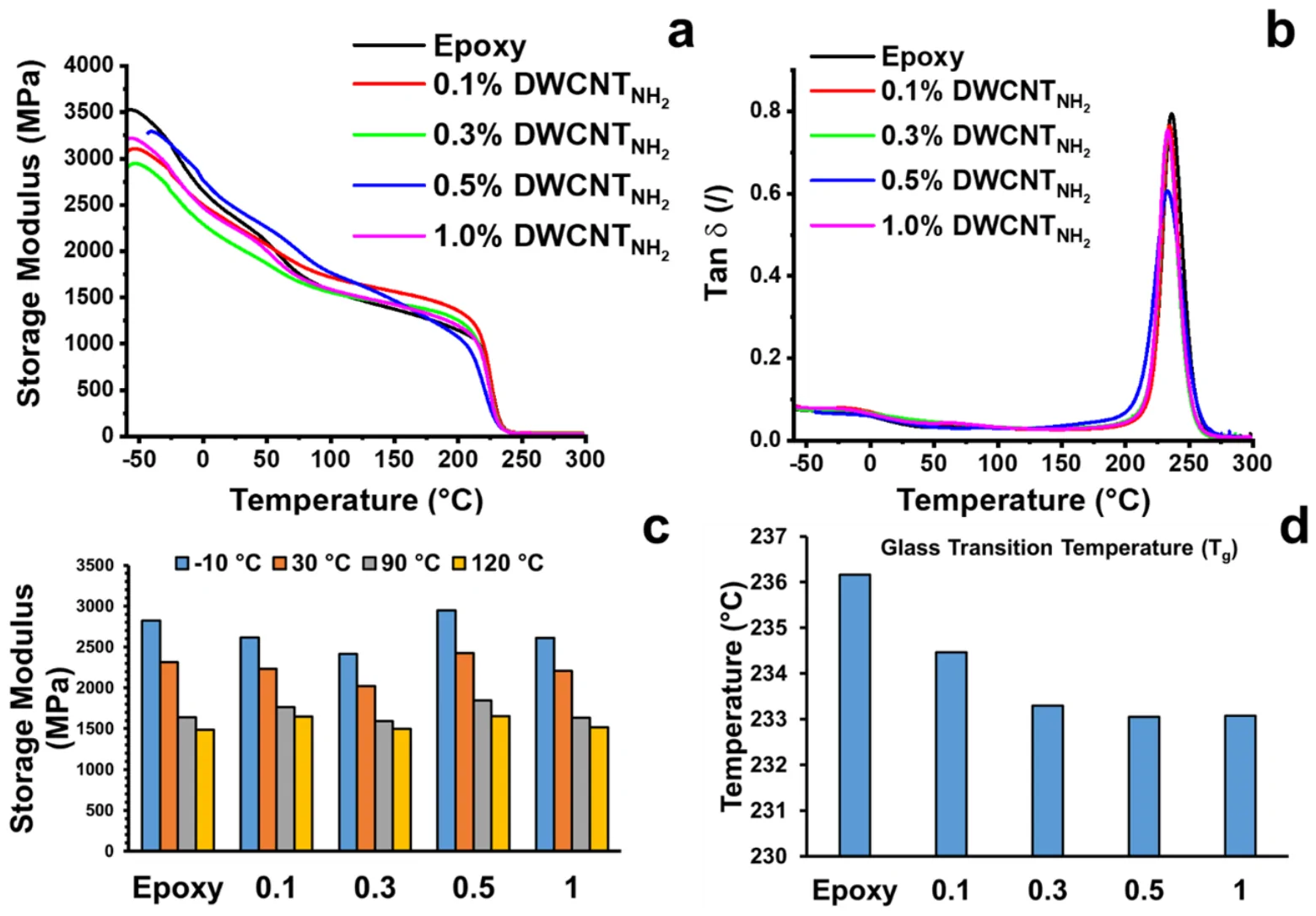
Dynamic mechanical analysis of the neat resin and nanocomposite samples: (**a**) temperature dependence of the storage modulus (E′); (**b**) evolution of the damping factor (tan δ) as a function of temperature; (**c**) storage modulus measured at selected temperatures (−10 °C, 30 °C, 90 °C, and 120 °C); (**d**) comparison of the maximum tan δ values for the investigated formulations.

Figure 10a,b report the temperature dependence of the storage modulus (E′) and damping factor (tan δ) for both the neat epoxy resin and the corresponding nanocomposites. As temperature increases, all materials exhibit the typical decrease in storage modulus associated with the progressive softening of the polymer network. For the unfilled resin, the α-relaxation process, corresponding to the glass transition, occurs at approximately 236 °C. The incorporation of nanoparticles influences both the viscoelastic stiffness and the damping behavior of the system. In particular, an increase in filler content leads to a gradual shift of the tan δ maximum toward lower temperatures, as shown in Figure 10d. Although the overall trend of the storage modulus remains relatively unaffected by nanoparticle addition, the internal organization of the material appears to undergo significant modifications, especially when examined at different temperature levels (Figure 10c and inset of Figure 10d). The glass transition phenomenon is governed by a combination of intermolecular and intramolecular interactions within the polymer network. Consequently, any factor affecting the curing process, including the presence of nanofillers or low-molecular-weight species, can alter the dynamic mechanical response of the material. Additional information regarding molecular relaxation can be obtained from the shape and intensity of the tan δ peaks. In the case of the neat epoxy resin, the tan δ curve between 200 and 260 °C is characterized by a single relaxation peak, indicating a relatively homogeneous distribution of relaxation mechanisms. A similar relaxation profile is observed for the nanocomposites, suggesting that the introduction of nanoparticles does not substantially modify the relaxation spectrum. However, a progressive reduction in glass transition temperature is clearly detected as the filler concentration increases. Since all nanocomposite specimens were prepared under the same conditions and subjected to an identical curing schedule, the observed changes can reasonably be attributed to the effect of the nanofiller on the polymer matrix. The variation in T_g_ results from the competition between two opposing mechanisms. On the one hand, nanofiller incorporation generally reduces the extent of cure attained under the same processing conditions, leading to a lower crosslink density than that of the neat resin [5].

On the other hand, the presence of high-aspect-ratio carbon nanotubes restricts the mobility of nearby polymer chains, which tends to increase T_g_ [70,71,72,73]. The combined action of these counteracting effects produces only a modest decrease in the glass transition temperature, typically limited to a few degrees. Nevertheless, the results indicate that the disruption of the crosslinking process caused by the filler plays the dominant role. This interpretation is consistent with the findings presented in Figure 9 and discussed in the preceding section, where evidence of a slight reduction in network development was already observed for the nanofilled systems.

### 3.4. Electrical Properties

**Figure 11:**
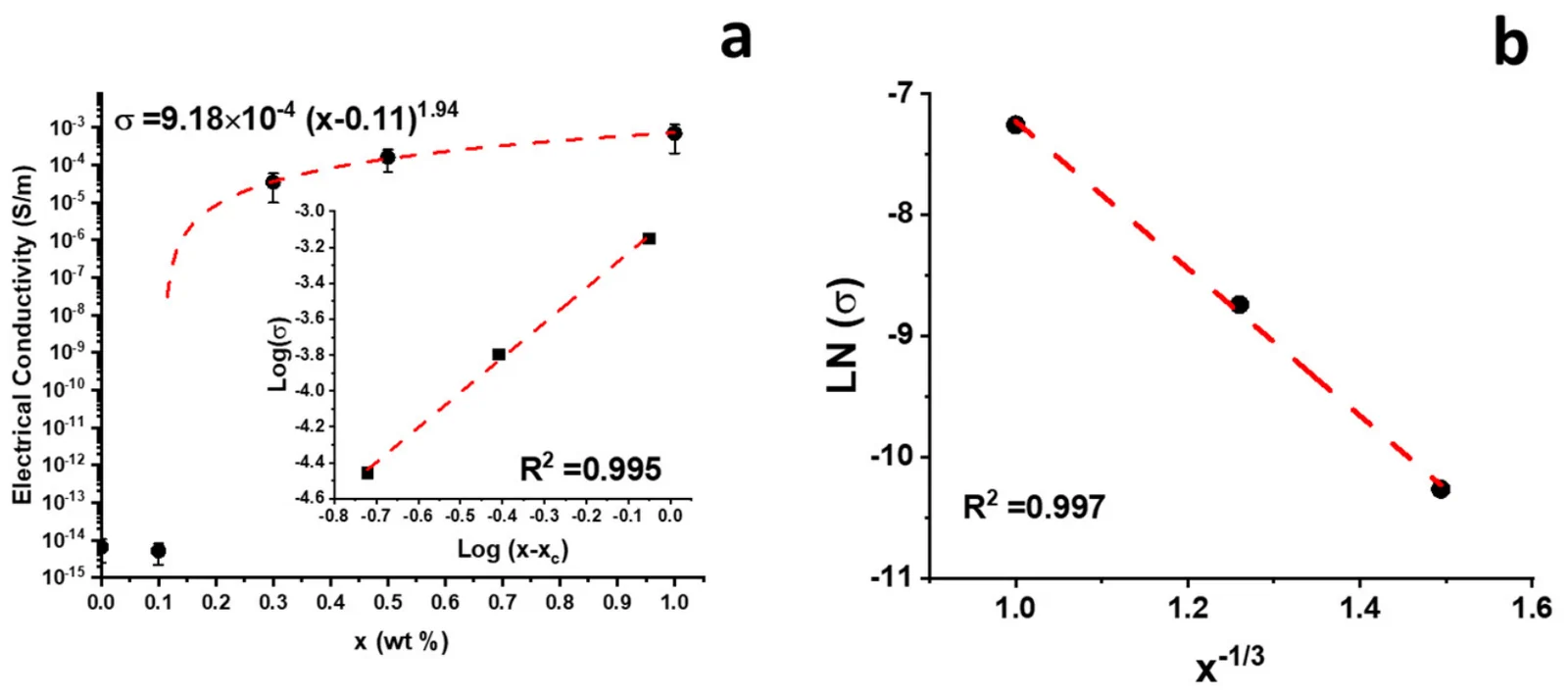
(**a**) DC electrical conductivity of the nanocomposite systems plotted against the weight fraction of DWCNTNH_2_. The inset shows the scaling behavior of conductivity above the electrical percolation threshold, reported as log(σDC) versus log(x − x_c_). (**b**) Plot of ln(σDC) as a function of x^−1/3^ for specimens containing filler contents higher than the EPT. Experimental data (markers) were fitted using Equations (10)–(12), as indicated by the dashed lines.

The DC electrical performance of the fabricated composites was assessed by evaluating their electrical conductivity (σ), electrical percolation threshold (EPT), the characteristic parameters governing the percolation model, and the prevailing charge transport mechanism. The dependence of the room-temperature DC volume conductivity on the DWCNTNH_2_ concentration (wt%) is presented in Figure 11a,b. As the conductive filler loading approaches the percolation threshold, the electrical conductivity increases dramatically compared with that of the neat resin. In particular, a composite containing only 1.0 wt% DWCNTNH_2_ exhibits a DC conductivity of approximately 5 × 10^−4^ S/m.

The electrical response of the investigated systems can be explained using the principles of percolation theory, which is commonly applied to polymer composites containing conductive particles dispersed in an insulating matrix. When the concentration of the conductive filler exceeds a critical level, known as the electrical percolation threshold (EPT), a significant change in the conduction mechanism occurs. At this point, isolated conductive domains become interconnected, forming continuous pathways throughout the material. As a consequence, charge carriers can move more efficiently across the composite, leading to a dramatic increase in electrical conductivity compared with the neat polymer. The establishment of this conductive network is responsible for the transition from insulating to conductive behavior and typically results in conductivity values that are several orders of magnitude higher than those of the unfilled matrix. Conversely, below the EPT, conductive pathways remain discontinuous, and the electrical response is dominated by the insulating matrix, resulting in conductivity values on the order of a few pS/m, as expected for dielectric materials. The percolation equation can describe the conductivity evolution above the percolation threshold:

(10) $$\sigma =\sigma_{0}{\left(x-x\right)}^{t}$$

where *σ* (S/m) represents the electrical conductivity of the nanocomposite, *x* is the nanotube weight fraction, *x_c_* is the percolation threshold, while *σ*_0_ (S/m) and *t* are the proportionality constant and the critical exponent of the percolation model, respectively. For fitting purposes, Equation (10) can be expressed in logarithmic form:

(11) $$\log\left(\rho \right)=\log\left(\sigma_{0}\right)+t\cdot log\left(x-x_{c}\right)$$

which corresponds to the linear expression (y = A + t,x), where A, i.e., $\log\left(\sigma_{0}\right),$ is the intercept and *t* is the slope of the linear relationship between the logarithm of the composite conductivity and the logarithm of the excess filler concentration above the percolation threshold. The log–log representations shown in the insets of Figure 11a were used to determine the characteristic percolation parameters of the investigated nanocomposites [70,74]. The electrical percolation threshold (EPT) was found to lie between 0.1 and 0.3 wt%, which is consistent with values typically reported for polymer composites reinforced with one-dimensional conductive nanofillers, including carbon nanotubes (CNTs) and carbon nanofibers (CNFs) [70,72]. Such a low percolation threshold indicates that an electrically continuous pathway is established even at very low filler concentrations through the formation of an interconnected conductive network between adjacent nanostructures. The precise location of the percolation threshold is influenced by several parameters, including the geometry and aspect ratio of the nanofillers, their state of dispersion within the polymer matrix, and the processing and fabrication methods adopted for composite preparation. The same log–log fitting procedure also yields a critical exponent, *t*, of approximately 1.9, corresponding to the slope of the linear regression. This value closely matches the theoretical universal exponent (≈2) expected for three-dimensional percolation phenomena [70], indicating that the electrical transport behavior of the investigated materials follows the classical percolation theory. Previous studies on epoxy nanocomposites containing one-dimensional conductive fillers have shown that, once a homogeneous nanotube dispersion is achieved, and the filler concentration exceeds the percolation threshold, the DC electrical conductivity is primarily controlled by electron tunneling between neighboring conductive particles. In this regime, charge transport is dominated by single junction tunneling events, and the electrical conductivity can be described by the following relationship:

(12) $$ln\left(\sigma_{DC}\right)\propto x^{-1/3}$$

Accordingly, the linear dependence observed between the logarithm of the DC conductivity and *x*^−1/3^, as shown in Figure 11b, provides strong evidence that electron tunneling represents the dominant charge transport mechanism in the investigated nanocomposites [71]. In this regime, the electrical current is controlled by the potential barriers separating adjacent conductive fillers, which remain isolated by ultrathin layers of insulating polymer resin.

### 3.5. Water Uptake Properties

**Figure 12:**
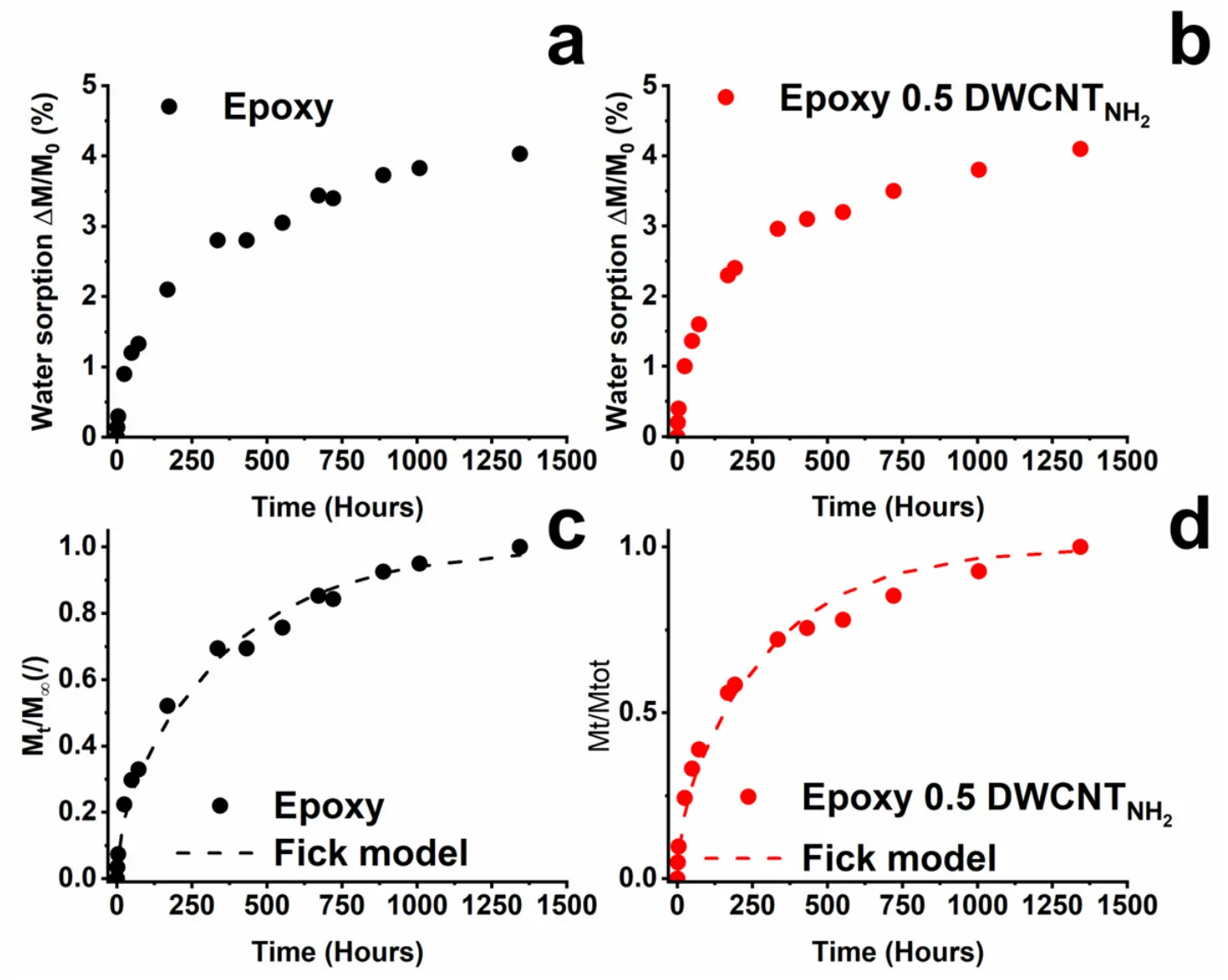
Water absorption behavior as a function of immersion time for (**a**) neat epoxy resin and (**b**) epoxy nanocomposite containing 0.5 wt% DWCNTNH_2_. Evolution of the normalized moisture uptake M_t_/M_∞_ with immersion time for (**c**) neat resin and (**d**) DWCNTNH_2_-reinforced epoxy composite (symbols represent experimental measurements, whereas solid lines correspond to the fitting results obtained using Fick’s diffusion model).

The moisture absorption of the specimens was determined by gravimetric measurements using a digital analytical balance with a resolution of 0.1 mg. Throughout the immersion experiment, the samples were removed from the water at predetermined time intervals, gently dried with lint-free paper to remove residual surface moisture and weighed immediately to minimize evaporation effects. The percentage of absorbed water was calculated using the following expression:

(13) $$\Delta M/M_{0}=100\cdot \frac{M_{t}-M_{0}}{M_{0}}$$

where *M_t_* is the specimen mass at immersion time (*t*), while *M*_0_ is the initial dry mass; the equilibrium water uptake (*M_∞_*) was taken as the maximum mass increase measured after 1340 h of immersion, corresponding to the plateau region of the *ΔM/M*_0_ curve. The water absorption behavior of the neat epoxy resin and the epoxy nanocomposite containing 0.5 wt% amino-functionalized carbon nanotubes is compared in Figure 12, while the corresponding experimental data are presented in Figure 12a and Figure 12b, respectively.

The moisture absorption results demonstrate that the incorporation of the nanofiller has only a minimal influence on the water uptake behavior of the epoxy system. The equilibrium moisture content changes only slightly, from approximately 4.03% for the unfilled resin to 4.10% for the nanocomposite, indicating that the presence of the carbon nanotubes does not significantly alter the overall moisture affinity of the matrix. A similarly limited effect is observed for the diffusion kinetics, which remain essentially comparable for both materials. The moisture diffusion coefficients were determined by fitting the experimental absorption curves with the analytical solution of Fick’s second law [75]. The fitting procedure employed the first 50 terms of the infinite-series solution expressed by:

(14) $$\frac{M_{t}}{M_{\infty }}=1-\sum_{0}^{\infty }\frac{8}{{\left(2n+1\right)}^{2}\pi^{2}}exp\left[-\frac{D{\left(2n+1\right)}^{2}\pi^{2}t}{l^{2}}\right]$$

where *M_t_* is the amount of water absorbed after an immersion time (*t*), *M_∞_* is the equilibrium moisture uptake, (*D*) is the diffusion coefficient, and (*l*) represents the specimen thickness. The agreement between the experimental measurements and the theoretical predictions obtained from Equation (14) is shown in Figure 12c,d, confirming that Fickian diffusion provides an appropriate description of the moisture transport process in both the neat epoxy and the nanotube-reinforced nanocomposite. The fitting procedure yielded diffusion coefficients of *D* = 2.18 × 10^−9^ cm^2^/s for the neat resin and *D* = 1.91 × 10^−9^ cm^2^/s for the filled system. The negligible effect on the water sorption properties can be attributed to the nature of the resin interfacial layer surrounding the nanotubes. NH_2_ functionalization promotes polyaddition reactions (see Figure 2 and Figure 9), resulting in a polymer chain structure remarkably similar to that formed by polyaddition of the neat resin. Consequently, only a slight change in the free volume is expected, which may account for the negligible changes observed in water diffusion and sorption behaviour. On the other hand, CNTs are highly susceptible to capillary effects and adsorption via hydrogen interactions at the interface; indeed, the presence of polar groups associated with nitrogen can favor such interactions.

As previously discussed, although the functionalized filler affects the crosslinking kinetics, its impact on the crosslinked network structure is minimal, as evidenced by the slight reduction in the glass transition temperature (T_g_). Given the low filler content introduced (up to 1 wt%), its presence does not significantly affect the free diffusion pathways of water molecules within the epoxy matrix, contributing negligibly to hydrogen interactions.

Consequently, the diffusion coefficient remains of the same order of magnitude. Furthermore, since the resin network structure is essentially unchanged, the concentration of hydroxyl groups (-OH) along the polymer chains (see Figure 2) also remains unchanged, thereby preserving the polarity of the matrix. As a result, the equilibrium water uptake remains comparable.

## 4. Conclusions

The present study demonstrates that amino-functionalized double-walled carbon nanotubes significantly influence both the curing process and the multifunctional properties of epoxy resins, even at very low filler contents. FTIR investigations, combined with Kamal’s kinetic model, showed that the nanotubes accelerate the initial epoxy–amine reaction, while the overall autocatalytic cross-linking mechanism remains essentially unchanged. Model-free isoconversional DSC analysis confirms the catalytic activity of the amino groups grafted on the nanotube surface, revealing a progressive decrease in the apparent activation energy in the intermediate and final stages of cross-linking as the nanotube concentration increases. The reaction between the functionalized nanotubes and the epoxy precursor promotes, most likely, the formation of an interphase with locally reduced crosslink density. This effect is reflected by the progressive reduction in the curing enthalpy, the slight decrease in glass transition temperature, and the modest reduction in the thermal degradation onset temperature, while the overall thermomechanical performance remains substantially preserved. From the functional viewpoint, the incorporation of DWCNTNH_2_ dramatically enhances the electrical conductivity of the epoxy matrix; conversely, the similarity of the diffusion coefficients and equilibrium sorption values indicates that the limited amount of functionalized nanotubes does not significantly modify either the free-volume characteristics or the polarity of the crosslinked epoxy network. Overall, amino-functionalized DWCNTs represent an effective multifunctional nanofiller capable of simultaneously accelerating epoxy curing, reducing the activation energy of network formation, and imparting electrical conductivity at extremely low filler concentrations. These characteristics make the investigated nanocomposites attractive candidates for multifunctional structural materials, electrically conductive adhesives, self-sensing components, and advanced electronic and aerospace applications.

## Figures and Tables

**Table 1:** Components of epoxy resin.

Product	Formulae	Supplier	Functional Group
DGEBA	** 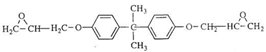 **	Sigma Aldrich	2
DDS	** 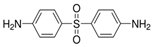 **	Sigma Aldrich	2 (4 active hydrogen atoms)

**Table 2:** Values of kinetic parameters of the obtained samples.

Sample	*K*_1_ (min^−1^)	*K*_2_ (min^−1^)	*n* (/)	*m* (/)	R^2^ (/)
Epoxy	0.0057	0.1542	1.89	0.47	0.999
Epoxy 0.5 DWCNTNH_2_	0.0242	0.1461	2.15	0.48	0.998

## Data Availability

The original contributions presented in this study are included in the article/Supplementary Material. Further inquiries can be directed to the corresponding author.

## Supplementary Materials

The following supporting information can be downloaded at: https://www.mdpi.com/article/10.3390/polym18172087/s1, Table S1: Chemical-physical characteristics of the DWCNTNH_2_, 2152 Grade (Data provided by Nanocyl S.A. upon request); Figure S1: TEM image of the DWCNTNH_2_, 2152 Grade (Provided by Nanocyl S.A. upon request); Figure S2: Thermogravimetric curve of functionalized DWCNTs.

## References

[1] Aparna A., Sethulekshmi A., Jayan J.S., Saritha A., Joseph K. (2021). Recent advances in boron nitride based hybrid polymer nanocomposites. Macromol. Mater. Eng..

[2] Ghassemi B., Estaji S., Mousavi S.R., Nemati Mahand S., Shojaei S., Mostafaiyan M., Arjmand M., Khonakdar H.A. (2022). In-depth study of mechanical properties of poly (lactic acid)/thermoplastic polyurethane/hydroxyapatite blend nanocomposites. J. Mater. Sci..

[3] Mokhtari Aghdami R., Mousavi S.R., Estaji S., Dermeni R.K., Khonakdar H.A., Shakeri A. (2022). Evaluating the mechanical, thermal, and antibacterial properties of poly (lactic acid)/silicone rubber blends reinforced with (3-aminopropyl) triethoxysilane-functionalized titanium dioxide nanoparticles. Polym. Compos..

[4] Mousavi S.R., Faraj Nejad S., Jafari M., Paydayesh A. (2021). Polypropylene/ethylene propylene diene monomer/cellulose nanocrystal ternary blend nanocomposites: Effects of different parameters on mechanical, rheological, and thermal properties. Polym. Compos..

[5] Guadagno L., Naddeo C., Raimondo M., Barra G., Vertuccio L., Russo S., Lafdi K., Tucci V., Spinelli G., Lamberti P. (2017). Influence of carbon nanoparticles/epoxy matrix interaction on mechanical, electrical and transport properties of structural advanced materials. Nanotechnology.

[6] Aliberti F., Vertuccio L., Longo R., Sorrentino A., Pantani R., Guadagno L., Raimondo M. (2025). Thermal, Mechanical, Morphological, and Piezoresistive Properties of Poly(ethylene-co-methacrylic acid) (EMAA) with Carbon Nanotubes and Expanded Graphite. Nanomaterials.

[7] De Vivo B., Lamberti P., Spinelli G., Tucci V., Guadagno L., Raimondo M., Vertuccio L., Vittoria V. (2013). Improvement of the electrical conductivity in multiphase epoxy-based MWCNT nanocomposites by means of an optimized clay content. Compos. Sci. Technol..

[8] Guadagno L., Aliberti F., Longo R., Raimondo M., Pantani R., Sorrentino A., Catauro M., Vertuccio L. (2023). Electrical anisotropy controlled heating of acrylonitrile butadiene styrene 3D printed parts. Mater. Des..

[9] Guadagno L., Longo R., Aliberti F., Lamberti P., Tucci V., Pantani R., Spinelli G., Catauro M., Vertuccio L. (2023). Role of MWCNTs loading in designing self-sensing and self-heating structural elements. Nanomaterials.

[10] Spinelli G., Lamberti P., Tucci V., Guadagno L., Vertuccio L. (2020). Damage monitoring of structural resins loaded with carbon fillers: Experimental and theoretical study. Nanomaterials.

[11] Aliberti F., Longo R., Sorrentino A., Raimondo M., Catauro M., Pantani R., Guadagno L., Vertuccio L. (2025). Strain sensors in creep tests. Sens. Actuators A Phys..

[12] Haddadi S.A., S.A. A.R., Mahdavian M., Arjmand M. (2021). Epoxy nanocomposite coatings with enhanced dual active/barrier behavior containing graphene-based carbon hollow spheres as corrosion inhibitor nanoreservoirs. Corros. Sci..

[13] Mousavi S.R., Estaji S., Raouf Javidi M., Paydayesh A., Khonakdar H.A., Arjmand M., Rostami E., Jafari S.H. (2021). Toughening of epoxy resin systems using core–shell rubber particles: A literature review. J. Mater. Sci..

[14] Mousavi S.R., Estaji S., Rostami E., Khonakdar H.A., Arjmand M. (2022). Effect of a novel green modification of alumina nanoparticles on the curing kinetics and electrical insulation properties of epoxy composites. Polym. Adv. Technol..

[15] Shekarchizadeh N., Jafari Nedoushan R., Dastan T., Hasani H. (2021). Experimental and numerical study on stiffness and damage of glass/epoxy biaxial weft-knitted reinforced composites. J. Reinf. Plast. Compos..

[16] Eker Y.R., Özcan M., Özkan A.O., Kırkıcı H. (2019). The influence of Al2O3 and TiO2 additives on the electrical resistivity of epoxy resin-based composites at low temperature. Macromol. Mater. Eng..

[17] Meng X., Yu H., Wang L., Wu X., Amin B.U. (2021). Recent progress on fabrication and performance of polymer composites with highly thermal conductivity. Macromol. Mater. Eng..

[18] Pełech I., Pełech R., Narkiewicz U., Kaczmarek A., Guskos N., Żołnierkiewicz G., Typek J., Berczyński P. (2020). Magnetic and electrical properties of carbon nanotube/epoxy composites. Mater. Sci. Eng. B.

[19] Zakaria M.R., Kudus M.H.A., Akil H.M., Thirmizir M.Z.M. (2017). Comparative study of graphene nanoparticle and multiwall carbon nanotube filled epoxy nanocomposites based on mechanical, thermal and dielectric properties. Compos. Part B Eng..

[20] Chen J., Han J., Xu D. (2019). Thermal and electrical properties of the epoxy nanocomposites reinforced with purified carbon nanotubes. Mater. Lett..

[21] Ghaleb Z., Mariatti M., Ariff Z. (2017). Synergy effects of graphene and multiwalled carbon nanotubes hybrid system on properties of epoxy nanocomposites. J. Reinf. Plast. Compos..

[22] Li A., Zhang C., Zhang Y.-F. (2017). Thermal conductivity of graphene-polymer composites: Mechanisms, properties, and applications. Polymers.

[23] Nouri-Borujerdi A., Kazemi-Ranjbar S. (2021). Thermal and electrical conductivity of a graphene-based hybrid filler epoxy composite. J. Mater. Sci..

[24] Wu S., Peng S., Wang C.H. (2018). Multifunctional polymer nanocomposites reinforced by aligned carbon nanomaterials. Polymers.

[25] Lin Y., Huang X., Chen J., Jiang P. (2017). Epoxy thermoset resins with high pristine thermal conductivity. High Volt..

[26] Wang Y., Weng G.J. (2017). Electrical conductivity of carbon nanotube-and graphene-based nanocomposites. Micromechanics and nanomechanics of Composite Solids.

[27] Han Z., Fina A. (2011). Thermal conductivity of carbon nanotubes and their polymer nanocomposites: A review. Prog. Polym. Sci..

[28] Mahanta N.K., Abramson A.R. Thermal conductivity of graphene and graphene oxide nanoplatelets. Proceedings of the 13th Intersociety Conference on Thermal and Thermomechanical Phenomena in Electronic Systems.

[29] Fogel M., Parlevliet P., Geistbeck M., Olivier P., Dantras E. (2015). Thermal, rheological and electrical analysis of MWCNTs/epoxy matrices. Compos. Sci. Technol..

[30] Imran K.A., Shivakumar K.N. (2018). Enhancement of electrical conductivity of epoxy using graphene and determination of their thermo-mechanical properties. J. Reinf. Plast. Compos..

[31] Roşu D., Caşcaval C., Mustątǎ F., Ciobanu C. (2002). Cure kinetics of epoxy resins studied by non-isothermal DSC data. Thermochim. Acta.

[32] Sbirrazzuoli N., Vyazovkin S., Mititelu A., Sladic C., Vincent L. (2003). A study of epoxy-amine cure kinetics by combining isoconversional analysis with temperature modulated DSC and dynamic rheometry. Macromol. Chem. Phys..

[33] Catalani A., Bonicelli M.G. (2005). Kinetics of the curing reaction of a diglycidyl ether of bisphenol A with a modified polyamine. Thermochim. Acta.

[34] Liang Y., Jing D., Bao-jun Q., Wen-fang S. (2006). Cure kinetics of DGEBA with hyperbranched poly (3-hydroxyphenyl) phosphate as curing agent studied by non-isothermal DSC. Chem. Res. Chin. Univ..

[35] Bae J., Jang J., Yoon S.H. (2002). Cure behavior of the liquid-crystalline epoxy/carbon nanotube system and the effect of surface treatment of carbon fillers on cure reaction. Macromol. Chem. Phys..

[36] Puglia D., Valentini L., Armentano I., Kenny J.M. (2003). Effects of single-walled carbon nanotube incorporation on the cure reaction of epoxy resin and its detection by Raman spectroscopy. Diam. Relat. Mater..

[37] Xie H., Liu B., Sun Q., Yuan Z., Shen J., Cheng R. (2005). Cure kinetic study of carbon nanofibers/epoxy composites by isothermal DSC. J. Appl. Polym. Sci..

[38] Xie H., Liu B., Yang H., Wang Z., Shen J., Cheng R. (2006). Thermal characterization of carbon-nanofiber-reinforced tetraglycidyl-4,4′-diaminodiphenylmethane/4,4′-diaminodiphenylsulfone epoxy composites. J. Appl. Polym. Sci..

[39] Xie H., Liu B., Yuan Z., Shen J., Cheng R. (2004). Cure kinetics of carbon nanotube/tetrafunctional epoxy nanocomposites by isothermal differential scanning calorimetry. J. Polym. Sci. Part B Polym. Phys..

[40] Abdalla M., Dean D., Robinson P., Nyairo E. (2008). Cure behavior of epoxy/MWCNT nanocomposites: The effect of nanotube surface modification. Polymer.

[41] Liu H.-K., Wang Y.-C., Huang T.-H. (2016). Moisture effect on mechanical properties of graphene/epoxy nanocomposites. J. Mech..

[42] Glaskova-Kuzmina T., Aniskevich A., Martone A., Giordano M., Zarrelli M. (2016). Effect of moisture on elastic and viscoelastic properties of epoxy and epoxy-based carbon fibre reinforced plastic filled with multiwall carbon nanotubes. Compos. Part A Appl. Sci. Manuf..

[43] Zulfli N.M., Bakar A.A., Chow W. (2013). Mechanical and water absorption behaviors of carbon nanotube reinforced epoxy/glass fiber laminates. J. Reinf. Plast. Compos..

[44] Lee B., Yang T., Wilusz E. (1996). Moisture effects on isobutylene-isoprene copolymer-based composite barrier. I: Moisture diffusion and detection. Polym. Eng. Sci..

[45] Garg M., Sharma S., Mehta R. (2016). Carbon nanotube-reinforced glass fiber epoxy composite laminates exposed to hygrothermal conditioning. J. Mater. Sci..

[46] Jana S., Zhong W.H. (2007). FTIR study of ageing epoxy resin reinforced by reactive graphitic nanofibers. J. Appl. Polym. Sci..

[47] De Vivo B., Guadagno L., Lamberti P., Raimo R., Sarto M.S., Tamburrano A., Tucci V., Vertuccio L. Electromagnetic properties of Carbon NanoTube/epoxy nanocomposites. Proceedings of the 2009 International Symposium on Electromagnetic Compatibility—EMC Europe.

[48] Guadagno L., De Vivo B., Di Bartolomeo A., Lamberti P., Sorrentino A., Tucci V., Vertuccio L., Vittoria V. (2011). Effect of functionalization on the thermo-mechanical and electrical behavior of multi-wall carbon nanotube/epoxy composites. Carbon.

[49] Simsek Y., Ozyuzer L., Seyhan A.T., Tanoglu M., Schulte K. (2007). Temperature dependence of electrical conductivity in double-wall and multi-wall carbon nanotube/polyester nanocomposites. J. Mater. Sci..

[50] Guadagno L., Vertuccio L. (2021). Resistive response of carbon nanotube-based composites subjected to water aging. Nanomaterials.

[51] Longo R., Guadagno L., Aliberti F., Schiavo L., Oliviero M., Sorrentino A., Fiorentino M., Vertuccio L. (2025). Exploitation of the tunneling effect for the development of self-sensing nanocomposite materials. Appl. Mater. Today.

[52] Morgan R.J., Mones E.T. (1987). The cure reactions, network structure, and mechanical response of diaminodiphenyl sulfone-cured tetraglycidyl 4, 4′ diaminodiphenyl methane epoxies. J. Appl. Polym. Sci..

[53] Vertuccio L., Calabrese E., D’Angelo A., Piccirillo A.M., Longo R. (2023). FTIR Analysis of the Curing Behaviors of Bi-Functional Epoxy Resin with Anhydride Based Hardener. Proceedings of the 6th International Conference on Design and Technologies for Polymeric and Composites Products (POLCOM 2022).

[54] Marquardt D.W. (1963). An algorithm for least-squares estimation of nonlinear parameters. J. Soc. Ind. Appl. Math..

[55] Maddams W. (1980). The scope and limitations of curve fitting. Appl. Spectrosc..

[56] Vertuccio L., Russo S., Raimondo M., Lafdi K., Guadagno L. (2015). Influence of carbon nanofillers on the curing kinetics of epoxy-amine resin. RSC Adv..

[57] Jungang G., Shigang S., Yangfang L., Deling L. (2004). Curing kinetics and thermal property characterization of bisphenol-F epoxy resin and DDS system. Int. J. Polym. Mater..

[58] Saeb M.R., Bakhshandeh E., Khonakdar H.A., Mäder E., Scheffler C., Heinrich G. (2013). Cure kinetics of epoxy nanocomposites affected by MWCNTs functionalization: A review. Sci. World J..

[59] Sbirrazzuoli N., Girault Y., Elégant L. (1997). Simulations for evaluation of kinetic methods in differential scanning calorimetry. Part 3—Peak maximum evolution methods and isoconversional methods. Thermochim. Acta.

[60] Turi E.A. (1997). Thermal characterisation of polymeric materials. Polym. Test..

[61] Vyazovkin S., Sbirrazzuoli N. (1996). Mechanism and kinetics of epoxy− amine cure studied by differential scanning calorimetry. Macromolecules.

[62] Vyazovkin S., Sbirrazzuoli N. (1999). Kinetic methods to study isothermal and non-isothermal epoxy-anhydride cure. Macromol. Chem. Phys..

[63] Vyazovkin S., Sbirrazzuoli N. (1999). Isoconversional method to explore the mechanism and kinetics of multistep epoxy cures. Macromol. Rapid Commun..

[64] Zvetkov V. (2001). Comparative DSC kinetics of the reaction of DGEBA with aromatic diamines.: I. Non-isothermal kinetic study of the reaction of DGEBA with *m*-phenylene diamine. Polymer.

[65] Vyazovkin S. (1997). Evaluation of activation energy of thermally stimulated solid-state reactions under arbitrary variation of temperature. J. Comput. Chem..

[66] Vyazovkin S. (2001). Modification of the integral isoconversional method to account for variation in the activation energy. J. Comput. Chem..

[67] Doyle C. (1962). Estimating isothermal life from thermogravimetric data. J. Appl. Polym. Sci..

[68] Sbirrazzuoli N., Vincent L., Vyazovkin S. (2000). Comparison of several computational procedures for evaluating the kinetics of thermally stimulated condensed phase reactions. Chemom. Intell. Lab. Syst..

[69] Wang S., Liang Z., Liu T., Wang B., Zhang C. (2006). Effective amino-functionalization of carbon nanotubes for reinforcing epoxy polymer composites. Nanotechnology.

[70] Bauhofer W., Kovacs J.Z. (2009). A review and analysis of electrical percolation in carbon nanotube polymer composites. Compos. Sci. Technol..

[71] Connor M.T., Roy S., Ezquerra T.A., Calleja F.J.B. (1998). Broadband ac conductivity of conductor-polymer composites. Phys. Rev. B.

[72] Guadagno L., Raimondo M., Vittoria V., Vertuccio L., Naddeo C., Russo S., Vivo B.D., Lamberti P., Spinelli G., Tucci V. (2014). Development of epoxy mixtures for application in aeronautics and aerospace. RSC Adv..

[73] Starkova O., Buschhorn S.T., Mannov E., Schulte K., Aniskevich A. (2013). Water transport in epoxy/MWCNT composites. Eur. Polym. J..

[74] Mohseni A., Hrymak A.N. (2026). A Review of Modeling Electrical Conductivity in Carbon-Filled Polymer Composites. Polymers.

[75] Crank J. (1979). The Mathematics of Diffusion.

