# Supplementary material for: Amino-Functionalized DWCNTs Tailor Curing Kinetics and Multifunctional Performance of Epoxy Nanocomposites
Source: Polymers (Basel). 2026 Aug 28;18(17):2087. doi: 10.3390/polym18172087 (PMC13568430; doi:10.3390/polym18172087)
Supplement: Supplementary file 1 [file polymers-18-02087-s001.zip › polymers-4486657-supplementary.pdf]

Amino-functionalized DWCNTs tailor curing kinetics and multifunctional performance of epoxy nanocomposites

Raffaele Longo[1], Liberata Guadagno[1], Marialuigia Raimondo[1], Francesca Aliberti[1], Michelina Catauro[2] and Luigi Vertuccio[2*]

[1]Department of Industrial Engineering, University of Salerno, Via Giovanni Paolo II, 132, 84084 Fisciano, SA, Italy; rlongo@unisa.it (R.L.); lguadagno@unisa.it (L.G.); mraimondo@unisa.it (M.R.); faliberti@unisa.it (F.A.)

[2]Department of Engineering, University of Campania "Luigi Vanvitelli", Via Roma 29, 813031 Aversa (CE), Italy; michelina.catauro@unicampania.it (M.C,); luigi.vertuccio@unicampania.it (L.V.)

Table S1. Chemical-physical characteristics of the DWCNTNH$_2$, 2152 Grade (Data provided by Nanocyl S.A. upon request)

Characterization DWCNTNH$_2$, 2152 Grade			
Property	Unit	Value	Method of Measurement
Average diameter	nm	4.7	HRTEM
Length	µm	several	TEM/SEM
Carbon Purity	%	> 90	TGA
Metal Oxide (impurity)	%	< 10	TGA
-NH$_2$ Functionalization	%	< 0.5	XPS

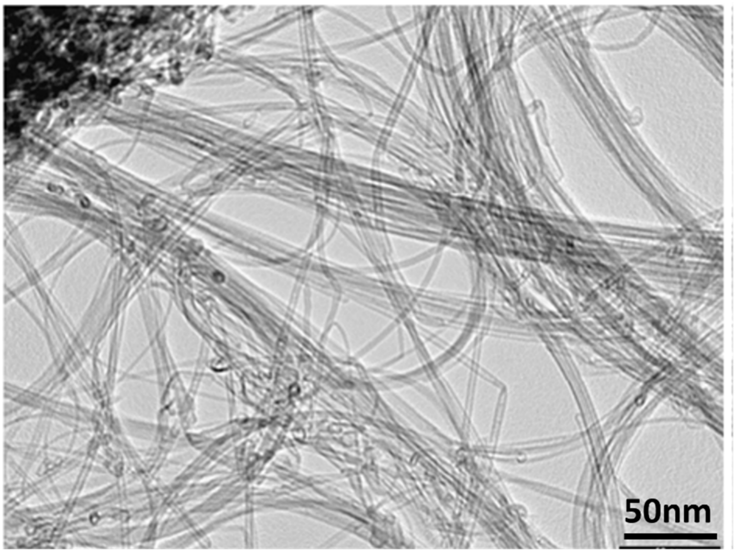

Figure S1. TEM image of the DWCNTNH$_2$, 2152 Grade (Provided by Nanocyl S.A. upon request)

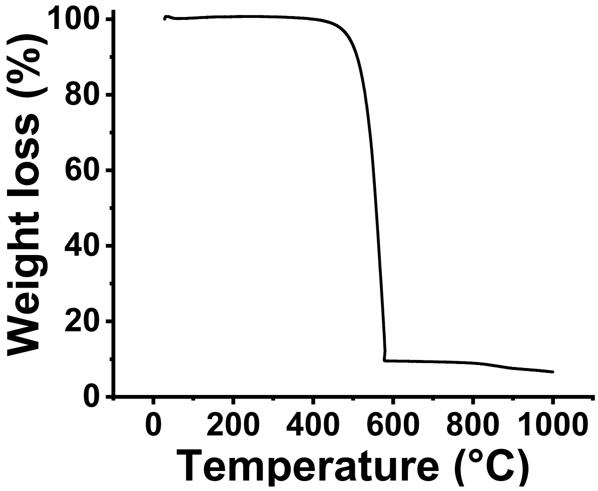

Figure S2. Thermogravimetric curve of functionalized DWCNTs.
